# LipL21 lipoprotein binding to peptidoglycan enables *Leptospira interrogans* to escape NOD1 and NOD2 recognition

**DOI:** 10.1371/journal.ppat.1006725

**Published:** 2017-12-06

**Authors:** Gwenn Ratet, Ignacio Santecchia, Martine Fanton d’Andon, Frédérique Vernel-Pauillac, Richard Wheeler, Pascal Lenormand, Frédéric Fischer, Pierre Lechat, David A. Haake, Mathieu Picardeau, Ivo G. Boneca, Catherine Werts

**Affiliations:** 1 Institut Pasteur, Unité Biologie et Génétique de la Paroi Bactérienne, Paris, France; 2 INSERM, équipe Avenir, Paris, France; 3 Institut Pasteur, Génopole (Protéopole), Paris, France; 4 Institut Pasteur, Unité de pathogenèse de Helicobacter, Paris, France; 5 Institut Pasteur, Hub Bioinformatique et Biostatistique, C3BI, USR 3756 IP CNRS, Paris, France; 6 Veterans Affairs Greater Los Angeles Healthcare System, Los Angeles, California, United States of America; 7 Department of Medicine, David Geffen School of Medicine at UCLA, Los Angeles, California, United States of America; 8 Department of Urology, David Geffen School of Medicine at UCLA, Los Angeles, California, United States of America; 9 Department of Microbiology, Immunology and Molecular Genetics, David Geffen School of Medicine at UCLA, Los Angeles, California, United States of America; 10 Institut Pasteur, Unité Biologie des Spirochètes, Paris, France; Medical College of Wisconsin, UNITED STATES

## Abstract

Leptospirosis is a widespread zoonosis, potentially severe in humans, caused by spirochetal bacteria, *Leptospira interrogans (L*. *interrogans)*. Host defense mechanisms involved in leptospirosis are poorly understood. Recognition of lipopolysaccharide (LPS) and lipoproteins by Toll-Like Receptors (TLR)4 and TLR2 is crucial for clearance of leptospires in mice, yet the role of Nucleotide Oligomerization Domain (NOD)-like receptors (NOD)1 and NOD2, recognizing peptidoglycan (PG) fragments has not previously been examined. Here, we show that pathogenic leptospires escape from NOD1 and NOD2 recognition both *in vitro* and *in vivo*, in mice. We found that leptospiral PG is resistant to digestion by certain hydrolases and that a conserved outer membrane lipoprotein of unknown function, LipL21, specific for pathogenic leptospires, is tightly bound to the PG. Leptospiral PG prepared from a mutant not expressing LipL21 (*lipl21*^-^) was more readily digested than the parental or complemented strains. Muropeptides released from the PG of the *lipl21*^-^ mutant, or prepared using a procedure to eliminate the LipL21 protein from the PG of the parental strain, were recognized *in vitro* by the human NOD1 (hNOD1) and NOD2 (hNOD2) receptors, suggesting that LipL21 protects PG from degradation into muropeptides. LipL21 expressed in *E*. *coli* also resulted in impaired PG digestion and NOD signaling. We found that murine NOD1 (mNOD1) did not recognize PG of *L*. *interrogans*. This result was confirmed by mass spectrometry showing that leptospiral PG was primarily composed of MurTriDAP, the natural agonist of hNOD1, and contained only trace amounts of the tetra muropeptide, the mNOD1 agonist. Finally, in transgenic mice expressing human NOD1 and deficient for the murine NOD1, we showed enhanced clearance of a *lipl21*^-^ mutant compared to the complemented strain, or to what was observed in NOD1KO mice, suggesting that LipL21 facilitates escape from immune surveillance in humans. These novel mechanisms allowing *L*. *interrogans* to escape recognition by the NOD receptors may be important in circumventing innate host responses.

## Author summary

Leptospirosis is a widespread zoonosis caused by spirochetal bacteria, *Leptospira interrogans (L. interrogans)*. *L*. *interrogans* are primarily extracellular pathogens although some reports suggest they may replicate within macrophages. In humans, leptospirosis can cause mild or severe disease, potentially leading to death, although rats or mice, which constitute the reservoir, are asymptomatic carriers. Host defense mechanisms involved in leptospirosis remain poorly understood. Toll-Like Receptor (TLR)2 and TLR4 are crucial for the clearance of *L*. *interrogans*, but the role of the cytosolic NOD receptors in leptospirosis is unknown. Here, we report that pathogenic leptospires escape the sensing of bacterial peptidoglycan through the NOD response. We found that an outer membrane lipoprotein of *L*. *interrogans* binds to and protects the peptidoglycan from degradation into muropeptides, thereby blocking signaling through NOD proteins. Moreover, in absence of this lipoprotein, the peptidoglycan of *L*. *interrogans* is properly sensed by human NOD1 but not by murine NOD1. This is due to the near absence of muramyl tetrapeptide, the murine NOD1 agonist, in the peptidoglycan of pathogenic leptospires. These novel mechanisms of NOD avoidance may facilitate the escape of leptospires from the innate immune system of their hosts.

## Introduction

*Leptospira interrogans (L*. *interrogans)* is a pathogenic spirochete responsible for leptospirosis, a globally distributed zoonosis. Infection with pathogenic leptospires leads to asymptomatic chronic carriage in kidneys of rodents, such as rats and mice, whereas in other animals, including humans, mild to severe acute disease may develop, with potentially fatal multiorgan system failure (Weil’s disease) [1]. *L*. *interrogans* colonizes a broad range of hosts, from arthropods to mammals and may have evolved strategies to escape their innate immune response.

The innate immune response relies on different humoral components, including the complement system, antimicrobial peptides and natural IgM that together participate in the elimination of pathogens. Mammalian innate immunity receptors of the Toll-like (TLR) and Nucleotide-binding Oligomerization Domain (NOD)-like (NLR) families are pattern recognition receptors (PRRs), that sense conserved microbial associated molecular patterns (MAMPs), such as bacterial lipopolysaccharide (LPS) or peptidoglycan (PG). Recognition of MAMPS by PRRs triggers a signaling cascade resulting in activation of NF-κB and other transcription factors, leading to secretion of pro-inflammatory cytokines, chemokines, antimicrobial peptides and ultimately to the attraction of phagocytes that clear pathogens [2].

Pathogenic leptospires have been shown to evade the human complement system through various mechanisms [3]. In addition, we have shown that the sensing of *L*. *interrogans* LPS by the TLR4 receptor is a crucial determinant that accounts for the difference in outcome of leptospirosis between mice and humans. Indeed, leptospiral LPS has atypical structural features [4], escapes human TLR4 recognition [5], and is unexpectedly recognized by TLR2 in human cells [6]. However, the lipid A moiety of the leptospiral LPS is sensed by murine TLR4 [5]. Consequently, mice deficient for TLR4 (TLR4KO) are sensitive to leptospirosis, while parental wild type (WT) mice are resistant to the infection [7, 8]. Interestingly, we demonstrated deleterious inflammation in infected mice deficient for both TLR4 and TLR2 (TLR2/4DKO) and MyD88KO mice, deficient for the main adaptor of TLRs. The question arose as to whether this TLR-independent inflammation could originate from leptospiral stimulation of other innate immune receptors, such as the cytosolic NLR receptors, encompassing both the inflammasome receptors (NLRP) and the NOD receptors that recognize bacterial PG. Our previous work showed that the NLRP3 inflammasome is synergistically stimulated by *L*. *interrogans* LPS and by a second cell wall component, the glycolipoprotein. This inflammasome response was dependent on both TLR2 and TLR4, and could therefore not account for the TLR-independent inflammation [7, 9, 10]. Consequently, we chose to examine whether pathogenic leptospires are recognized by the cytosolic NOD1 and NOD2 receptors.

PG is a macromolecule of the cell envelope forming a mesh-like layer that determines the shape of the bacterium and withstands the internal turgor pressure of the cell. The PG polymer is composed of antiparallel chains of two repeated acid sugars, N-acetyl glucosamine (GlcNAc) and N-acetyl muramic acid (MurNAc). A peptide of 3 to 5 amino acids is linked to each muramic acid monomer and the peptides from adjacent chains are cross-linked.

The human receptors NOD1 and NOD2 recognize muropeptides, which are fragments of PG released upon digestion by bacterial hydrolases. These enzymes participate physiologically in bacterial biosynthesis, recycling and remodeling of PG. Muropeptides can also be released by host enzymes, such as the lysozyme that hydrolyses the ß-1,4 bond between MurNAc and GlcNAc, leading to cleavage of PG and lysis of the bacteria. Lysozyme is also a very potent cationic antimicrobial peptide, which destabilizes bacterial membranes [11]. The NOD2 receptor recognizes Muramyl dipeptide (MDP), present in PG of all bacteria [12], whereas the human NOD1 receptor recognizes the Muramyl tri-peptide with a meso-diaminopimelic acid (*meso*DAP) in the third position (MurTriDAP) [13, 14], usually found in PG of Gram-negative bacteria.

Leptospiral PG, which confers the helical shape of *L*. *biflexa* [15], is characterized by a *meso*DAP in the third position of the peptide [16]. Other spirochetes, such as *Borrelia* or *Treponema* spp. have an ornithine residue at this position [16]. Accordingly, *Borrelia* spp. have been shown to be recognized by NOD2 together with TLR2, but not by NOD1 [17]. A 1996 study showed that leptospiral PG was able to stimulate human cells to secrete pro-inflammatory cytokines [18]. Together, these data suggest that the leptospiral PG could be sensed by both NOD1 and NOD2. Herein, we examined recognition of PG of pathogenic leptospires by NOD1 and NOD2 receptors. Unexpectedly, we found that an abundant lipoprotein, LipL21, of previously unknown function, is tightly bound to the PG and blocks its digestion by hydrolases *in vitro*, thus impairing the recognition of leptospiral muropeptides by NOD receptors. This peculiar mechanism would constitute a novel bacterial strategy to escape the NOD innate immune response.

## Results

### Absence of NOD1 and NOD2 does not affect the course of leptospirosis

We recently generated a bioluminescent strain of pathogenic *L*. *interrogans* serovar Manilae (MFLum1), allowing for tracking leptospires by live imaging (IVIS) [10]. In mice intraperitoneally (IP) infected with MFLum1, IVIS revealed a biphasic infection. During the first week post infection (acute phase), leptospires disseminate and replicate in blood, then disappear from the circulation and appear in urine. In the second week, leptospires progressively reappear, restricted to the kidney. One month post infection, leptospires are stably established in kidney (chronic phase), for a life-time colonization [10].

**Fig 1:**
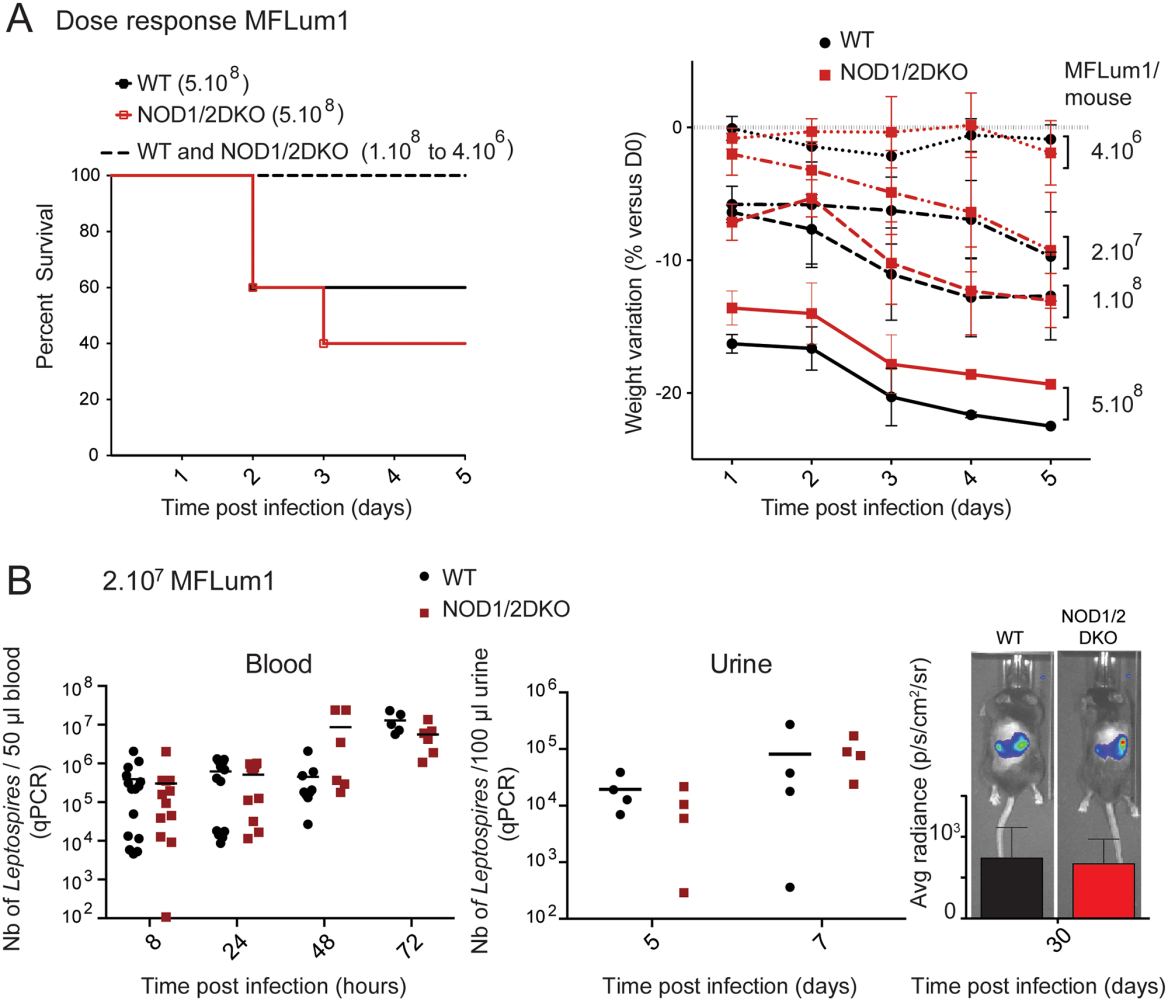
NOD1 and NOD2 receptors do not affect *L*. *interrogans* dissemination. (A) Survival curves (left panel) and weight variation (right panel) of C57BL6/J (WT) and NOD1 and NOD2 deficient (NOD1/2DKO) mice after intraperitoneal infection with 4 different doses (from 5.10^8^ to 4.10^6^ leptospires/mouse) of MFLum1, a bioluminescent strain of *L*. *interrogans* serovar Manilae L495 (weekly passage 14). Survival curves and weight loss were established within the 5 days following the infection, corresponding to the acute phase of the disease. Percentage of weight loss is represented as the mean ± SEM of individual mice compared to their initial weights before infection at day 0 (D0). For each dose, n = 5 WT and n = 5 NOD1/2 DKO mice. (B) Bacterial loads in WT and NOD1/2DKO mice IP infected with a sub-lethal dose of 10^7^ bioluminescent *L*. *interrogans* (MFLum1). Bacterial loads were measured by qPCR in blood at 8, 24, 48 and 72 hours post infection, and in urine at 5 and 7 days post infection, and by live imaging (IVIS), 1 month post infection. The graphs represent a compilation of 3 independent experiments with n = 3 to 6 mice for the blood panel, 1 experiment for the urine panel and 1 experiment representative of 3 with n = 4 mice for the IVIS panel. (A),(B). Statistics are not indicated as no significant differences were found at any time points between WT and NOD1/2DKO mice.

To assess the role of NOD1 and NOD2 in the clearance of pathogenic leptospires, C57BL6/J WT mice and NOD1/NOD2 double deficient mice (NOD1/2DKO) were IP infected with different doses of *L*. *interrogans* strain MFLum1 (weekly passage 14) starting from its known median lethal dose in WT mice of 5.10^8^ to the lower dose of 4.10^6^ bacteria per mouse (Fig 1A), using 5 times decreasing doses. Clinical observation and weight measures were performed daily. Lethality was only observed at the higher dose of 5.10^8^ bacteria (Fig 1A) and was equivalent in WT and NOD1/2DKO mice. Indeed, 2 out of 5 WT and 3 out of 5 NOD1/2DKO mice died or were euthanized at day 2 or 3 post infection. Only mice presenting both clinical signs of acute leptospirosis (ruffled fur, prostration, hypothermia) and that lost more than 20% of their initial weight were euthanized. Considering all doses, weight losses, in accordance with clinical symptoms of leptospirosis, were proportional to the bacterial dose used for infection, and did not show any significant differences between WT and NOD1/2DKO mice (Fig 1A).

### Absence of NOD1 and NOD2 does not affect *L*. *interrogans* dissemination

To get a more subtle insight in a potential role of NOD receptors against leptospires, bacterial loads were measured by qPCR in blood samples at 8, 24, 48 and 72 hours post infection, in urine samples at 5 and 7 days post infection, and by live imaging (IVIS) one month post infection (Fig 1B), representing acute and chronic phase of leptospirosis. Results show that bacterial loads in blood or urine of WT and NOD1/2DKO mice were similar either at the acute phase in blood or at the chronic phase in kidneys.

Together these results suggest that the NOD receptors do not play any major role in the control of leptospirosis in mice. Previous work evidenced that saprophytic *Leptospira* possess an atypical PG [15], a feature that could account for the absence of recognition through the NOD1/2 receptors. Therefore, we decided to examine the PG of the pathogen *L*. *interrogans*.

### The PG from *L*. *interrogans* is difficult to digest in muropeptides

**Fig 2:**
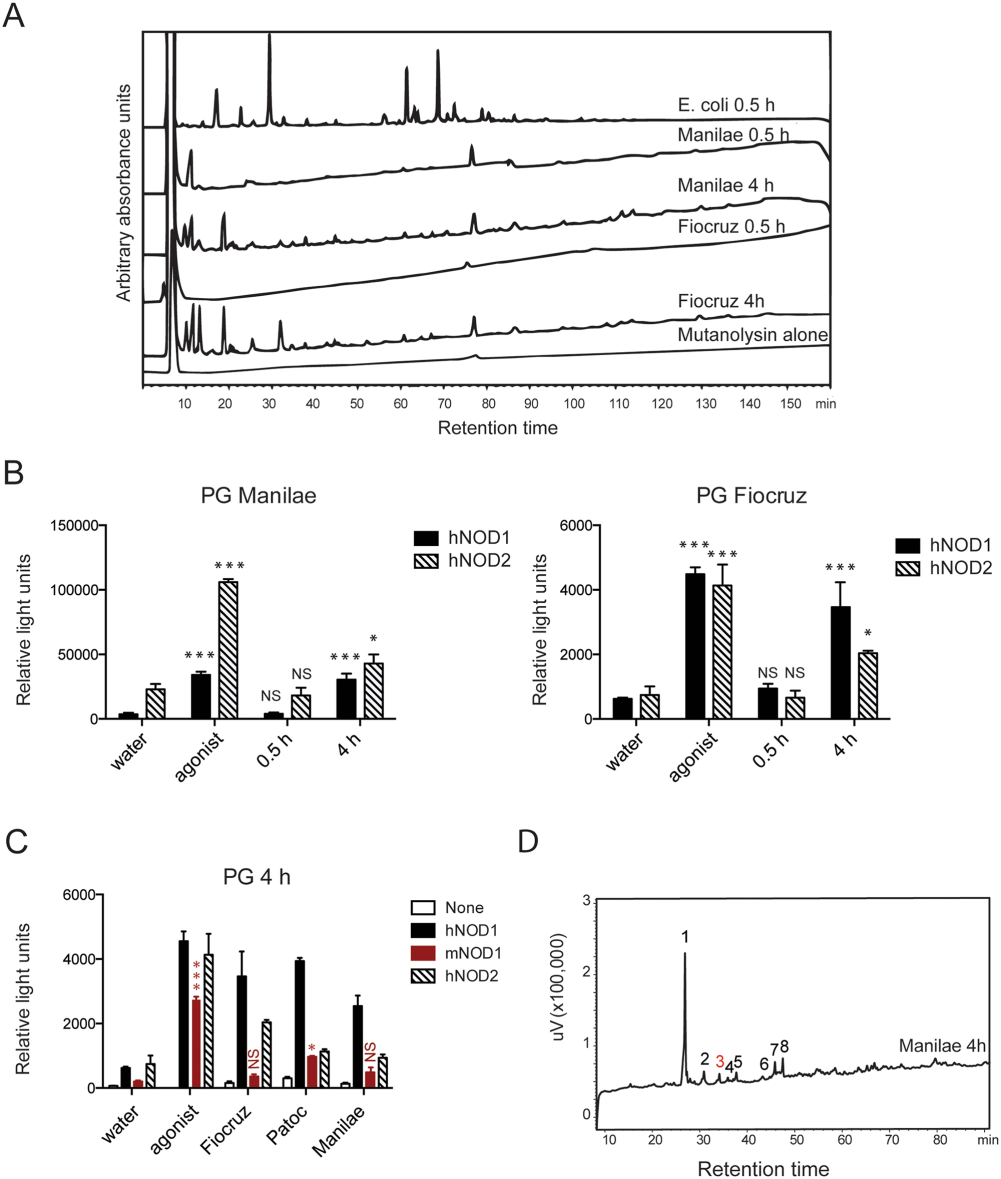
Digested *L*. *interrogans* PG is recognized by human NOD1 and NOD2 but barely by murine NOD1. (A) HPLC separation profiles of leptospiral muropeptides. Peptidoglycan (PG) extraction protocol influences the digestion of leptospiral PG into muropeptides. PG obtained from *L*. *interrogans* Manilae L495 and Fiocruz L1-130, extracted with 2 different protocols, called respectively 0.5 h and 4 h were digested by mutanolysin before HPLC. Commercial *E*. *coli* PG (Sigma) was used as the positive control for the mutanolysin digestion. Mutanolysin without PG (mutanolysin alone) was included as a control. (B) Leptospiral muropeptides signal through human NOD1 (hNOD1) and NOD2 (hNOD2). Six μg of PG 0.5 h and 4 h of *L*. *interrogans* Manilae (left panel) and Fiocruz (right panel), the NF-κB-luciferase reporter and NOD1 or NOD2 plasmids were co-transfected in HEK 293T cells. MurTriDAP (MTP) and MDP (100 nM) were used as positive controls (agonists) for NOD1 and NOD2 activation, respectively. Luciferase activity was measured 24 h after transfection. Data are expressed as the mean ± SEM of triplicates of relative light units, representing luciferase activity normalized with respect to β-galactosidase activity and are representative of 5 experiments. For each transfection, the unpaired *t* test was used to compare each condition to the negative control (water). A *p* value < 0.05 was considered significant. *p* values: *p < 0.05, ***p < 0.001. NS: non significant. (C) PG of pathogenic strains (Manilae L495 / Fiocruz L1-130) does not signal through murine NOD1. HEK293T cells were co-transfected with 10 μg of PG from Manilae L495, Fiocruz L1-130 strains or *L*. *biflexa* strain Patoc, prepared with the 4h SDS boiling protocol, along with the NF-κB-luciferase reporter (None) and human (h)NOD1, hNOD2 or murine NOD1 (mNOD1) plasmids. For each transfection, cells were stimulated with water as negative control, or with 100 nM of MurTriDAP, FK156, or MDP (agonists) as positive controls for hNOD1, mNOD1 and hNOD2 activation, respectively. Luciferase activity was measured 24 h after transfecting the cells. Data are expressed as the mean ± SEM of triplicates of relative light units representing luciferase activity normalized with respect to β-galactosidase activity and are representative of 3 experiments. The unpaired *t* test was used to compare the recognition of each PG by mNOD1 transfected cells (in red) to the negative control (water). A *p* value < 0.05 was considered significant. *p* values: ***p < 0.001. NS: non significant. For clarity, statistics relative to hNOD1 and hNOD2, already showed in panel (A), are not indicated. (D) HPLC analysis of the PG of *L*. *interrogans* strain Manilae prepared with the 4 h protocol and treated with chemotrypsin before digestion with mutanolysin. The numbered peaks were collected, and analyzed by mass spectrometry. The corresponding composition is in Table 1. The peak numbered 3 in red corresponds to GM4, the murine NOD1 agonist.

To study the PG of *L*. *interrogans*, we first purified the PG from the pathogenic Fiocruz L1-130 and Manilae L495 strains with a protocol routinely used in our laboratory for Gram-negative bacteria (boiling of bacteria 0.5 h in SDS, (hereafter 0.5 h)) [19]. We used mutanolysin, a hydrolase with the same specificity as lysozyme, to digest PG and obtain muropeptides that were analyzed by HPLC. The flat HPLC profiles obtained (Fig 2A) suggested that digestion of leptospiral PG by mutanolysin failed, presumably as a result of inappropriate PG purification protocol. In comparison, PG from *E*. *coli*, treated with the same enzyme, was properly digested into muropeptides that could be resolved by HPLC (Fig 2A).

Interestingly, we observed that multiple freeze/thaw cycles of the leptospiral PG increased digestion by mutanolysin (S1A Fig), suggesting that protein contaminants may have impaired the digestion. Therefore we changed the protocol and increased the boiling time of *L*. *interrogans* in SDS from 0.5 to 4 h (hereafter 4 h), as previously performed for purification of the PG of saprophytic strains of leptospires [15]. Under this condition, the PG of the pathogenic Fiocruz L1-130 and Manilae L495 strains were better digested into muropeptides (Fig 2A). Similar results were obtained with the PG of another pathogenic *L*. *interrogans* (serovar Icterohaemorraghiae strain Verdun) (S1B Fig). PG preparations from different serovars of *L*. *interrogans* presented roughly similar muropeptides profiles (Fig 2A and S1B Fig).

### Muropeptides from *L*. *interrogans* PG stimulate human NOD1 and NOD2 receptors

Next, to study the reactivity of NOD receptors towards the leptospiral muropeptides, the reporter HEK293T cells expressing the human NOD1 or NOD2 receptors were stimulated with PG of *L*. *interrogans* strains Fiocruz L1-130 and Manilae L495 prepared from 0.5 h and 4 h protocols (Fig 2B). Interestingly, the PGs 0.5 h did not stimulate the cells, consistent with the results obtained with NOD1/2DKO mice. In contrast, both PG purified from the 4 h protocol stimulated human NOD1 (hNOD1) and to a lesser extent the human NOD2 (hNOD2) (Fig 2B), showing that the 4 h protocol allowed for the release of muropeptides from PG of *L*. *interrogans*. Altogether these results suggest that the PG of *L*. *interrogans* is protected from degradation by hydrolases, which may impair recognition by NOD receptors.

### PG from *L*. *interrogans* is not recognized by the murine NOD1

Because NOD1 and NOD2 proteins were not involved in the clearance of leptospires in mice, although human NOD1 and NOD2 recognized leptospiral PG, we next compared the murine and human NOD1 responses towards the 4 h PG preparation of both pathogenic strains (Fig 2C). Interestingly, recognition by the murine NOD1 (mNOD1) receptor of both leptospiral PG was very limited compared to the hNOD1 recognition (Fig 2C). By contrast, we found that the PG from the saprophytic *L*. *biflexa* Patoc strain was recognized by the mNOD1 (Fig 2C). We did not check the murine NOD2 since we did not previously observe any species specificity of NOD2 recognition between human and mice.

### Mass spectrometry analysis reveals an atypical muropeptides profile

To better understand the discrepancy observed between murine versus human NOD1 recognition that could be due to specific muropeptide profiles of the leptospiral PG, individual muropeptide peaks were identified by HPLC and mass spectrometry, obtained using the 4 h PG of the Manilae L495 strain, after mutanolysin digestion (Fig 2D and Table 1). An atypical profile was observed, characterized by a single dominant peak (numbered 1 on the HPLC profile Fig 2D) corresponding to muramyl tripeptide (GM3), the human NOD1 agonist. Peak 2 corresponds to deacetylated GM3. Interestingly, the peak numbered 3 corresponding to the muramyl tetra peptide (GM4) was very limited, and GM5 was not found (Fig 2D and Table 1). Since the specificity of recognition of the NOD1 receptor has been shown to differ between human and mouse, with GM4 being the agonist of mNOD1 whereas GM3 is the agonist of hNOD1 [20, 21], this result suggests that leptospiral PG possesses only a very limited amount of muropeptides able to trigger the mNOD1 recognition, although it possesses a fair amount of hNOD1 agonists. The same observations were also true for the Verdun and Fiocruz L1-130 strains (S1B and S1C Fig). The PG from the Patoc strain (S1D Fig) slightly differed from the pathogenic strain with more dimers (S1D Fig), as already shown in a previous study also indicating that GM4 represented 4% of the Patoc’s muropeptides, while the pathogenic L495 strain had only 2% of GM4 [15]. We could not confirm these proportions since our digestion only allowed visualization and mass spectrometry analyzes of major peaks (S1C Fig). Together, these results suggest that leptospires evolved strategies to escape from NOD responses, first by impairing the degradation of its PG into muropeptides, and additionally by peculiarities of the structure of their PG, for example by a reduced amount of the GM4 muropeptide, the murine NOD1 agonist.

**Table 1:** Identification of muropeptides of L495 PG (neutral molecular mass) corresponding to the HPLC chromatogram shown in Fig 2C.

*Peak ID*	*Muropeptide*	*Measured mass*	*Theoretical mass*
**1**	GM3	870.3741	870.3706
**2**	GM3-Ac	828.3635	828.3600
**3**	GM4	941.4116	941.4077
**4**	GM-GM3(NH_2_)	1348.5564	1348.5505
**5**	GM-GM3(NH_2_)–Ac	1306.5448	1306.5399
**6**	GanhM4+3	1313.5722	1313.5722
**7**	GM3+GM4	1793.7759	1793.7677
**8**	GM3+GM4	1793.7751	1793.7677

Peak ID corresponds to the numbered peaks in Fig 2C. The proposed structural compositions were confirmed by MS/MS fragmentation. G, *N*-acetylglucosamine; M, *N*-acetylmuramic acid; anhM, anhydro-*N*-acetylmuramic acid; 3, L-Ala-isoGlu-*meso*DAP; 4, L-Ala-isoGlu-*meso*DAP-D-Ala; NH_2_ signifies amidation of *meso*DAP;–Ac, signifies deacetylation of one *N*-acetylglucosamine residue (*i*.*e*. glucosamine). Peaks 7 and 8 have the same measured mass. The difference in retention time most likely reflects alternative conformations of the dimer that could not be differentiated by MS analysis.

### Absence of NOD1 and NOD2 receptors does affect the clearance of the *L*. *biflexa* strain

*L*. *biflexa* are different from the pathogenic *L*. *interrogans* strains, since they have a reduced generation time (4h versus 18h), are sensitive to complement and never cause disease [1]. We previously showed using live imaging of a bioluminescent derivative of the *L*. *biflexa* Patoc strain (PFLum7) that it is mostly cleared within 24h post IP injection [10]. Since in contrast to pathogenic strains, Patoc’s PG was recognized by the murine NOD1, we tested whether NOD1/2DKO mice could be less effective to clear this strain. Therefore, we compared the clearance of 5.10^8^ PFLum7 in WT and NOD1/2DKO (S2 Fig), but we did not evidence any significant difference in the kinetics of clearance of the bacteria, suggesting that NOD1 and NOD2 receptors do not participate in the recognition of the *L*. *biflexa* Patoc strain.

### LipL21 lipoprotein is associated with *L*. *interrogans’* PG

**Fig 3:**
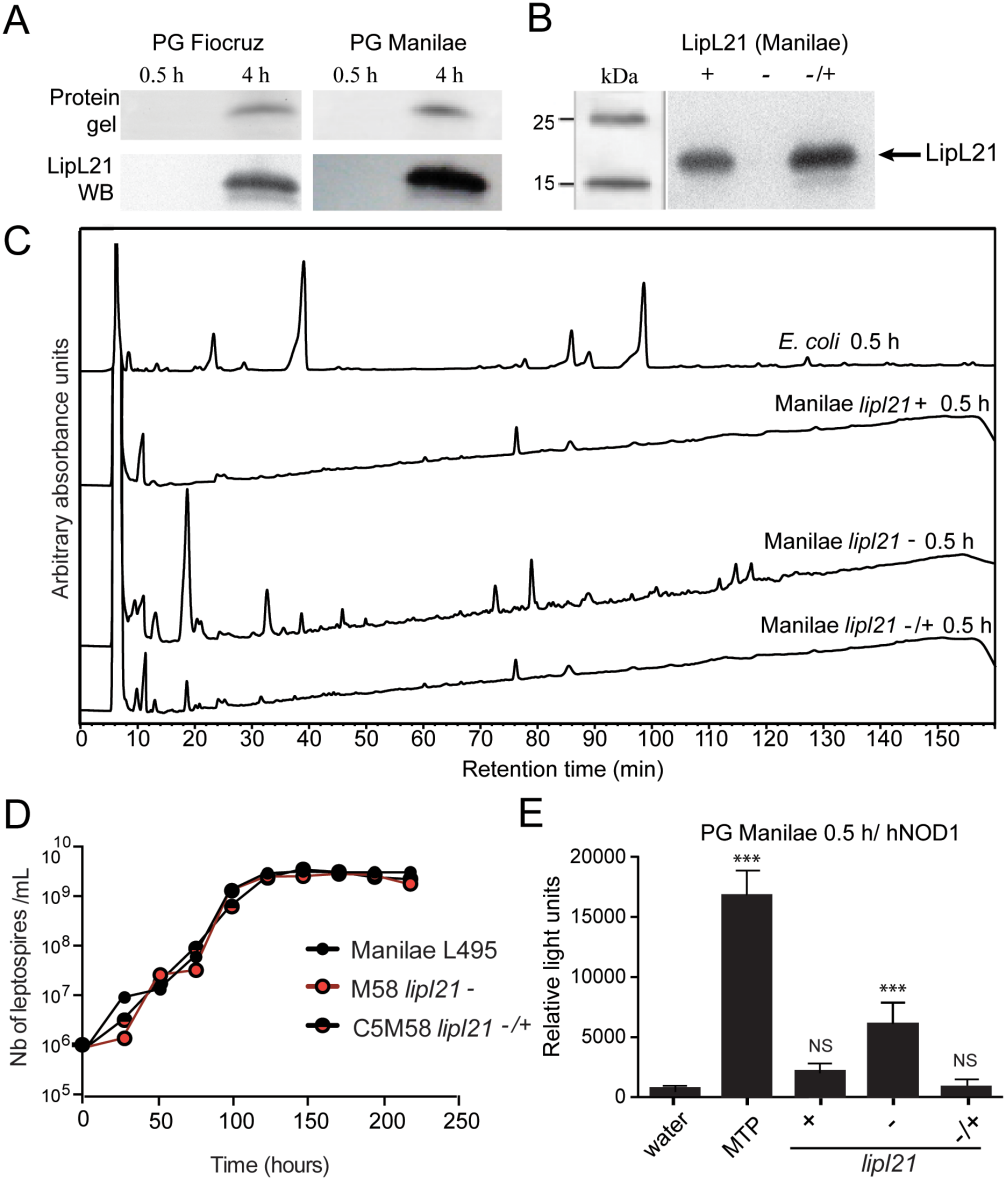
The LipL21 lipoprotein impairs leptospiral peptidoglycan digestion into muropeptides. (A) PG 0.5 h and PG 4 h from *L*. *interrogans* Manilae L495 and Fiocruz L1-130 strains, were loaded on coomassie-stained gels (upper panels; protein gels), revealing a 21-kDa protein corresponding to the LipL21. The protein specificity was checked by immunodetection using LipL21 antiserum (lower panels; western blot, (WB)). (B) LipL21 expression checked by immunodetection on bacterial extracts from *L*. *interrogans* Manilae L495 (*lipl21*^+)^, the (*lipl21*^-^) M58 mutant and the complemented C5M58 strain (*lipl21*^-/+^). (C) HPLC separation profiles of muropeptides after digestion by mutanolysin of *L*. *interrogans* Manilae *lipl21*^+^, *lipl21*^-^ and *lipl21*^-/+^ peptidoglycans, extracted with the 0.5 h protocol. As positive control for the mutanolysin digestion, *E*. *coli* PG was extracted with the leptospiral 0.5 h protocol. D) Growth curves of *L*. *interrogans* Manilae *lipl21*^+^, *lipl21*^-^ and *lipl21*^-/+^ strains in EMJH medium at 28°C, with agitation. (E) Muropeptides or 6 μg of PGs extracted from the parental Manilae strain (*lipl21*+), the LipL21 mutant (*lipl21*^-^) and the complemented strain (*lipl21*^-/+^) were co-transfected with the reporters and NOD1 plasmids in HEK293T cells. 24 h after transfection, luciferase activity was measured. MurTriDAP (MTP) (100 nM), the NOD1 agonist was used as positive control and water as negative control (water). Data are expressed as the mean ± SEM of relative light units representing luciferase activity normalized with respect to β-galactosidase activity. This graph is representative of 3 independent experiments. The unpaired *t* test was used to compare the recognition of each PG by hNOD1 transfected cells to water as a negative control (none). A *p* value < 0.05 was considered significant. *p* values: *** *p* < 0.001. Non significant differences for the + and -/+ PG are not indicated.

We suspected that some proteins may be linked to the PG and could have been degraded by the 4 h boiling procedure. For this reason, PG preparations from both the 0.5 h and 4 h boiling protocol were electrophoresed on a polyacrylamide gel and stained with Coomassie blue. Since PG is a macromolecule and does not enter the gel, and because muropeptides are not stained by Coomassie blue, only proteins could be detected. Indeed, one band of approximately 20 kDa was present in the Fiocruz L1-130 and Manilae L495 PGs prepared with the 4 h protocol but was missing in the PG prepared with the 0.5 h protocol (Fig 3A). This suggested that boiling *L*. *interrogans* in SDS for 0.5 h was insufficient to detach the 20 kDa protein, which was retained within the PG and did not enter the gel. The 20 kDa band analyzed after trypsin digestion was identified by mass spectroscopy as the LipL21 leptospiral lipoprotein, which was checked by immunoblotting with a polyclonal antibody directed against LipL21 (Fig 3A). LipL21 has been shown to be an abundant outer membrane surface exposed lipoprotein, of unknown function, conserved among pathogenic leptospires, and expressed during infection [22]. BLAST searches with the LipL21 protein sequence from *L*. *interrogans* serovar Manilae showed that LipL21 is restricted to Spirochetes, and present in all *Leptospira* species (spp.). ClustalX2 alignments of selected protein sequences show that LipL21 sequences are distributed in three statistically well-supported subgroups. Interestingly, the three sequence subgroups follow the distribution of the three pathotypes that have been described for *Leptospira* spp.: pathogenic, intermediate pathogenic and non-pathogenic (S3 Fig).

### PG from *L*. *interrogans* mutant in LipL21 is easily digested and signals through hNOD1

One mutant (M58) in the *lipl21* gene, and its complemented partner (C5M58) were obtained after transposon mutagenesis performed on *L*. *interrogans* serovar Manilae strain L495, as described [23]. The expression of LipL21 was checked by immunodetection in the crude extracts. The *lipl21* mutant (hereafter named *lipl21*^-^) was devoid of LipL21, and the complemented strain (called *lipl21*^*-/+*^), expressed slightly more LipL21 than the parental L495 strain (Fig 3B). The growth rate of *lipl21*^-^ was equivalent to the parental L495 and complemented strains (Fig 3D). Interestingly, in contrast to the PG from both parental and *lipl21*^*-/+*^ complemented strains, the PG from the *lipl21*^-^ mutant prepared with the 0.5 h protocol was effectively digested by mutanolysin and the pattern of muropeptide peaks was observed in HPLC (Fig 3C). In correlation with these results and by comparison with PG from both parental and complemented strain, PG from the *lipl21*^-^ mutant also clearly signaled through hNOD1 (Fig 3E). Taken together, these results suggest that LipL21 binding to PG impairs the degradation of PG and subsequent signaling through NOD receptors.

### Heterologous expression of LipL21 in *E*. *coli* alters the PG digestion and NOD signaling

**Fig 4:**
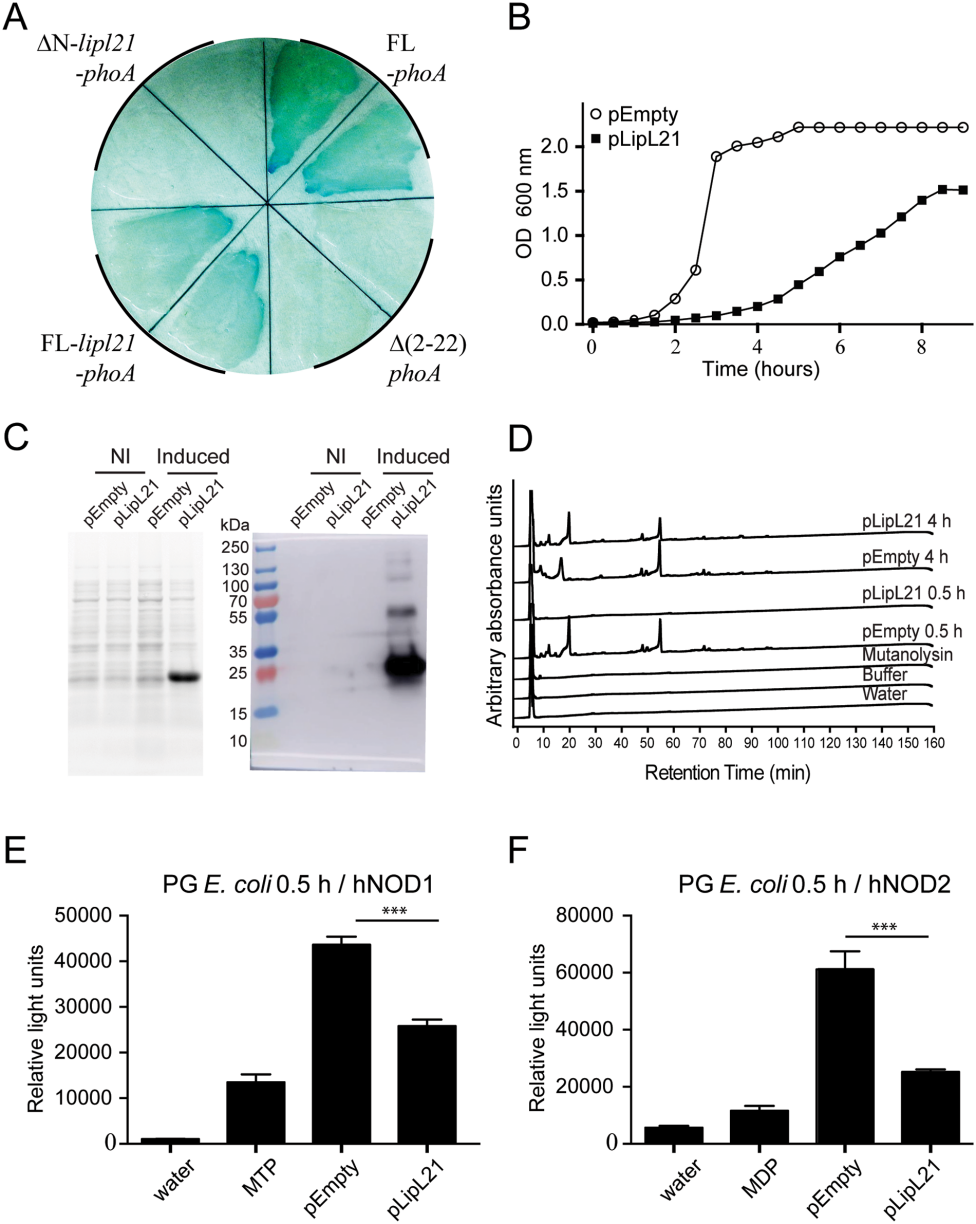
Expression of LipL21 impairs *E*. *coli* peptidoglycan digestion into muropeptides and recognition by NOD receptors. (A) Color phenotypes of strains expressing alkaline phosphatase (phoA) derivatives and grown as colonies on agar with chloramphenicol and 5-bromo-4-chloro-3-indoyl-phosphate (XP). FL-*phoA*, Δ(2–22)phoA, FL-*lipl21-phoA* and ΔN-*lipl21-phoA* correspond respectively to *E*. *coli* (BTH_101_) expressing the full length phoA (positive control), *E*. *coli* expressing phoA without signal peptide (negative control), *E*. *coli* expressing the full length LipL21 fused with Δ(2–22)*phoA* and *E*. *coli* expressing the LipL21 without signal peptide fused with Δ(2–22)*phoA*. (B) Growth curves of BL-21 Rosetta-2 *E*. *coli* expressing the LipL21 lipoprotein (pLipL21) or not (pEmpty) in the pRSFDuet-1 vector, without IPTG induction. (C) Crude bacterial extracts of BL-21 Rosetta-2 *E*. *coli* with empty pASK-IBA6 vector (pEmpty) or LipL21 expressing vector (pLipL21), induced by anhydrous-Tetracyclin or not (NI) and migrated on 12% acrylamide gel. Stain free coloration (left) and immunodetection (right) using a LipL21 antiserum. The Rainbow marker ladder gives an indication for the LipL21size.(D) HPLC separation profiles of muropeptides after digestion with mutanolysin. Each peptidoglycan was extracted with both the 0.5 h and 4 h protocols from BL-21 Rosetta-2 *E*. *coli* expressing the LipL21 lipoprotein (pLipL21) or not (pEmpty), after induction with anhydrous-Tetracyclin. (E) and (F) *E*. *coli* PGs, extracted from the 0.5 h protocol, were used to stimulate HEK 293T cells expressing human NOD1 (E) or NOD2 (F). HEK 293T cells were co-transfected with 6 μg of PG or 100 nM of muropeptides controls (MTP for NOD1 and MDP for NOD2, along with the reporter constructions and NOD1 or NOD2 expression plasmids. Luciferase activity was measured 24 h after transfection. Cells left untreated with muropeptides were used as negative control (water). Data are expressed as the mean of triplicates ± SEM of relative light units representing luciferase activity normalized with respect to β-galactosidase activity. The graph shown is representative of 3 equivalent experiments. The unpaired *t* test was used to compare the recognition of PGs prepared from *E*. *coli* with the empty vector (pEmpty) to the PG prepared from *E*. *coli* expressing LipL21 (pLipL21). A *p* value < 0.05 was considered significant. *p* values: *** *p* < 0.001. For clarity, statistics comparing each PG or muropeptide control to the water treated cells have not been indicated but are all significant with at least p<0,05.

An alternative way to assess the role of LipL21 in the protection of PG is to express the recombinant protein in a heterologous organism and study the capacity of extracted PG to be digested into muropeptides in the presence of hydrolases. Since LipL21 is an outer membrane (OM) lipoprotein of *L*. *interrogans* [22], it requires appropriate secretion across the inner membrane, to be acylated, and to reach its final OM location. To first verify that LipL21 was correctly targeted to the periplasm in *E*. *coli*, we used an alkaline phosphatase (PhoA) secretion assay. After its synthesis, the pro-PhoA is translocated across the plasma membrane towards the periplasm, with cleavage of its N-terminal sequence and maturation of disulfide bonds, essential for activity. Thus, if PhoA reaches the periplasm, it is fully active, and its activity can be monitored using a synthetic substrate (5-Bromo-4-chloro-3-indolyl phosphate, XP) that turns blue upon PhoA-dependent cleavage. We constructed a series of 4 plasmids, one carrying the full Pro-PhoA gene (positive control), one with the 5’ sequence corresponding the targeting signal removed (Δ(2–22)*phoA* gene, negative control, no secretion) and two plasmids carrying the full length LipL21-PhoA fusion gene, and the ΔN-LipL21-PhoA fusion gene, where the N-terminal sequence of LipL21 had been removed. *E*. *coli* carrying plasmids with these constructions were tested for PhoA activity, and results show that the LipL21-PhoA fusion is targeted to the periplasm (blue staining upon XP and IPTG addition), while the ΔN-LipL21-PhoA fusion is not (no blue staining), compared to control plasmids, indicating that LipL21 is appropriately targeted to the periplasm in the *E*. *coli* heterologous context (Fig 4A). Moreover the LipL21-PhoA fusion protein was easily detected (S4 Fig), showing that LipL21 can be correctly expressed in *E*. *coli*.

However, contrary to the PhoA fusions, different attempts to express the freestanding LipL21 in various *E*. *coli* strains were unsuccessful. Using the BL-21 Rosetta-2 *E*. *coli* strain, expressing tRNAs corresponding to rare codons, we succeeded in obtaining transformants from the pRSF-duet1. However, even without the induction of the protein expression, the generation time of the BL-21 Rosetta-2 strain harboring the LipL21 expression plasmid (pLipL21) was impaired (135 min) compared to the control strain transformed with the empty plasmid (pEmpty) (24 min), suggesting a toxic effect of the LipL21 lipoprotein expression (Fig 4B).

For improved regulation of expression, the *lipl21* gene without its signal peptide was cloned in the tightly regulated pASK-IBA-6 vector, containing the signal peptide of OmpA to direct the protein to the periplasm. This allowed expression of LipL21 without deleterious effects on the bacterial growth. To limit toxicity, the protein expression was induced for only 3 hours in the BL-21 Rosetta-2 strain. After confirmation of LipL21 expression (Fig 4C), the PGs from *E*. *coli* strains, expressing or not the LipL21 protein, were prepared with both 0.5 h and 4 h protocols and digested with mutanolysin. The HPLC profiles showed that the PGs from *E*. *coli* with the empty plasmid prepared from the 0.5 h and 4 h protocols were equally well digested, whereas the PG prepared from the *E*. *coli* strain expressing LipL21 was not digested following the 0.5 h protocol, but required the 4 h boiling to be digested (Fig 4D), demonstrating that LipL21 could protect *E*. *coli* PG from digestion. These results mirror those obtained with *L*. *interrogans* PG, and demonstrate that LipL21 plays a similar function in the protection of PG in a heterologous context of *E*. *coli*.

Furthermore, the recognition by hNOD1 and hNOD2 of PG from *E*. *coli* expressing LipL21, prepared from the 0.5 h protocol, was reduced compared to the PG from the *E*. *coli* strain harboring the empty vector (Fig 4E and 4F), again mimicking the results obtained in *L*. *interrogans*. Taken together these results suggest that LipL21 binds to *meso*DAP containing PG and impairs the release of muropeptides, which in turn alleviates recognition of the bacterium by the innate immune receptors NOD1 and NOD2.

### The M58 mutant is avirulent, independently of the *lipL21*- mutation

**Fig 5:**
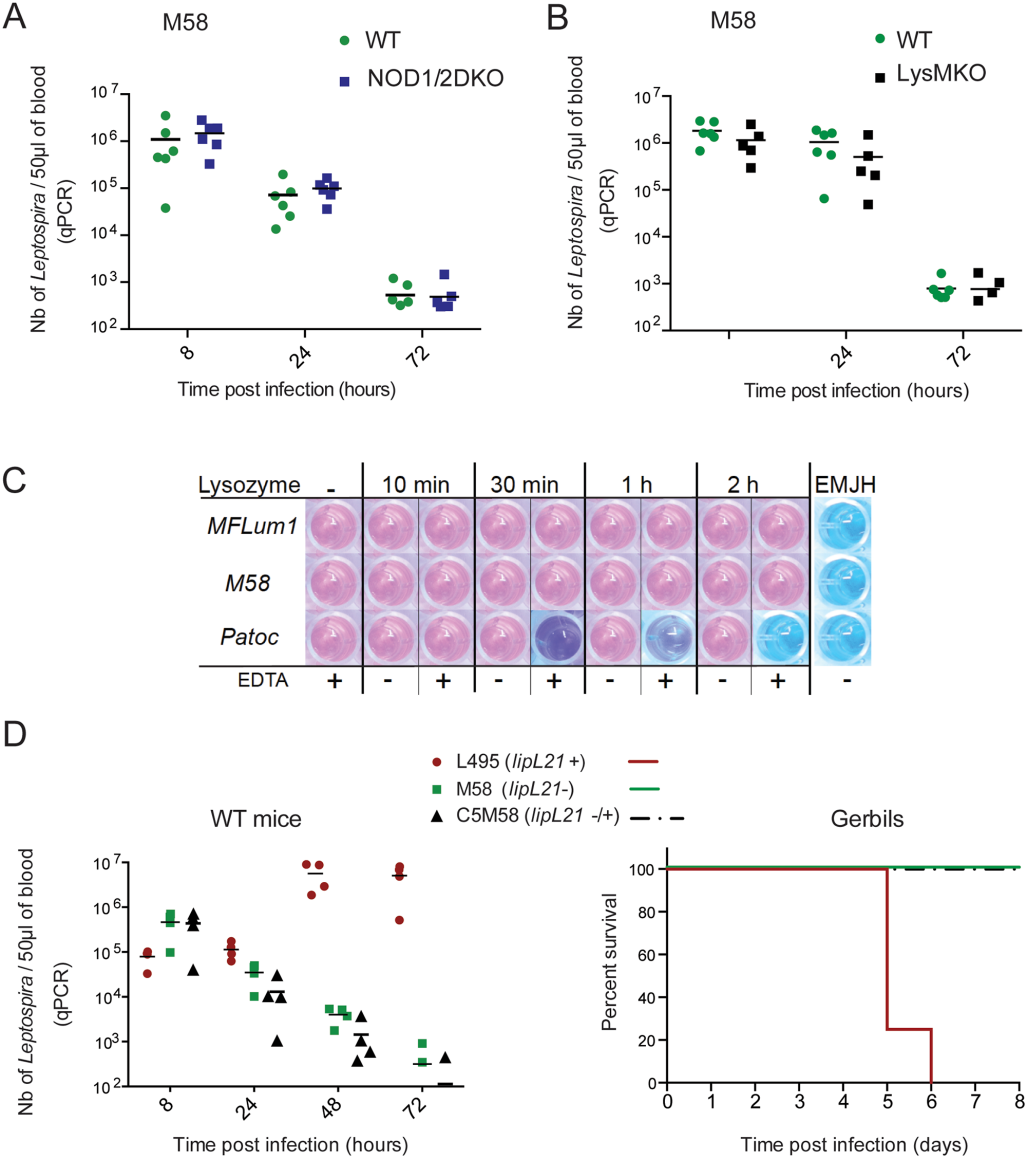
M58 is not virulent and the complementation does not restore virulence. (A)(B) M58 is not virulent in mice and the loss of virulence is not due to PG sensing. (A) C57BL6/J (WT) mice and mice deficient for both NOD1 and NOD2 receptors (NOD1/2DKO) and (B) FVB (WT) mice and mice deficient for lysozyme expressed in macrophages (LysMKO) were IP infected with 2.10^7^ M58 *lipl21*- mutant. Bacterial loads were measured by qPCR in blood at 8, 24 and 72 hours post infection. These experiments are representative of 2 equivalent experiments with n = 4 to 7 mice in each group. Statistics are not indicated as no significant differences were found at any time points between WT and NOD1/2DKO mice (A), or WT and LysMKO mice (B). (C) *L*. *interrogans* are not susceptible to lysozyme *in vitro*; leptospires were incubated at 28°C in presence of lysozyme and EDTA for the indicated times, then washed and further cultured for 5 days in EMJH medium. The Alamar blue dye was added and further incubated for 2 days. A pink color means that the bacteria are viable whereas the blue color indicates dead bacteria, reflecting the killing by lysozyme in presence of EDTA. Each plate included a positive control (bacteria with EDTA (+) and without lysozyme (-)), and a negative control (medium only). (D) The LipL21 complementation does not restore the virulence of M58 (*lipl21*-) in mice (left panel) nor in gerbils (right panel). C57BL6/J (WT) mice were IP infected with a sub-lethal dose of 10^7^ L495 strain or with 2.10^7^ of M58, the *lipl21*- mutant or C5M58, the complemented strain of M58 expressing LipL21 (*lipl21-/+*). Bacterial loads were measured by qPCR in blood at 8, 24, 48 and 72 hours post infection. This experiment is representative of 2 equivalent experiments with n = 4 to 7 mice in each group. Statistics are not indicated as no significant differences were found at any time points between M58 and C5M58 complemented strain. Survival curves of gerbils IP infected with 10^6^ bacteria/ ml in EMJH. n = 4 gerbils /group.

We infected WT and NOD1/2DKO mice with the mutant *lipl21*- strain (M58) and compared the kinetics of dissemination of the bacteria in blood in the first 3 days post infection. Instead of disseminating in the blood as observed for the virulent strain MFLum1 (Fig 1A), the *lipl21*- mutant was cleared from blood after 72 hours post infection in the WT mice, showing that the M58 strain had lost virulence (Fig 5A). No difference in clearance was observed in NOD1/2DKO compared to WT mice, suggesting that the lack of virulence was not linked to the PG sensing through mNOD1, which was expected, but also not through NOD2 (Fig 5A). For this reason we wondered whether the lack of virulence of the *lipl21*- mutant could be due to a higher sensitivity to lysozyme, a potent anti-microbial peptide that can also cleave the PG. Hence, we infected with M58 parental FVB (WT) mice and LysMKO mice, deficient for the lysozyme expressed in macrophages [24]. No difference of clearance of M58 was observed between WT and LysMKO mice (Fig 5B). Moreover, an *in vitro* test did not show any enhanced susceptibility to lysozyme of the *lipl21*- mutant compared to MFLum1, expressing LipL21 (Fig 5C). Altogether these results suggest that the loss of virulence of M58 is not linked to the muropeptides sensing by the NOD receptors nor to a better access of the PG to lysozyme.

We next infected WT mice with the M58 *lipl21*^-^ mutant or the complemented C5M58 *lipl21*^-/+^ strain. The complemented strain was also cleared after 3 days of infection in mice, showing that the LipL21 complementation did not restore the virulence (Fig 5D, left panel). We also did a lethal challenge in gerbils comparing the virulent MFLum1 to the *lipl21*^-^ mutant and complemented strain. M58 and C5M58 strains did not kill the gerbils, in contrast to the MFLum1 (Fig 5D, right panel). A whole DNA sequencing analysis of the M58 strain showed that beside silent single nucleotide polymorphisms (SNPs), 5 non synonymous SNPs were found in M58 compared to the parental L495 strain. 3 out of these 5 SNPs were found in an unknown ORF, 1 in a transposase gene and 1 in the *fliM* gene, coding for the flagellar motor switch, known to be involved in virulence [25] (Table 2). Those results suggest that the M58 mutant lost its virulence independently of the *lipl21* mutation, most probably in the course of the mutagenesis, and that those SNPs may be responsible for the loss of virulence.

**Table 2:** Missense non synonymous SNP mutations found by DNA sequencing of genome of M58 compared to the parental strain L495.

*Locus (MAGE)* *L*. *interrogans* Manilae L495	*Name*	*SNP*	*Function*
LMANv2_150152		T14N L30I L37F	Unknown
LMANv2_190001		S58P	Transposase
LMANv2_340043	*fliM*	T61P	Flagellar motor switch

### The *lipl21*- mutant is better cleared in humanized NOD1 mice

**Fig 6:**
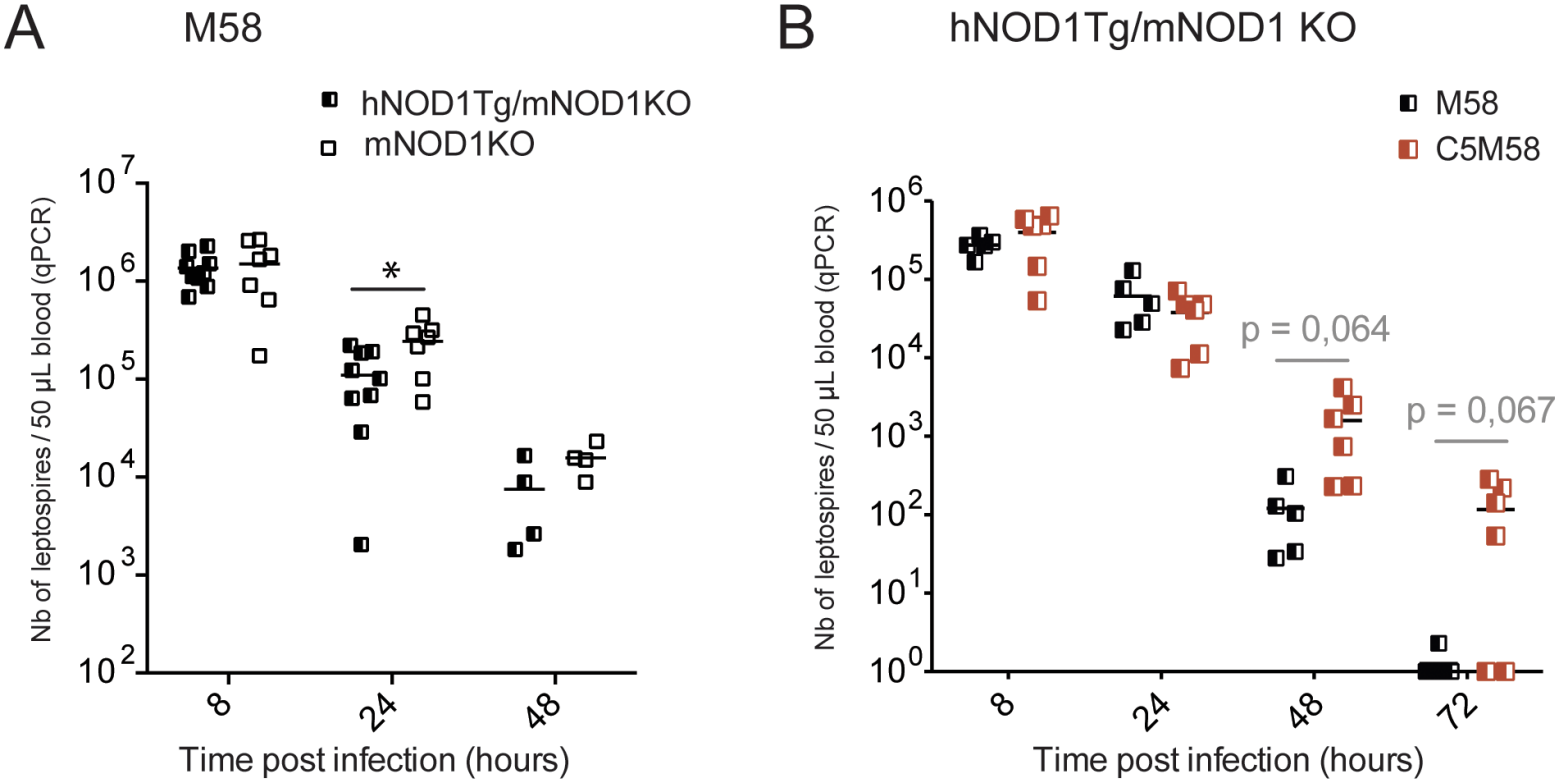
The human NOD1 receptor expressed in mice confers a slightly enhanced clearance of M58. (A) Transgenic C57BL6/J mice for the human NOD1 gene and deficient for NOD1 (hNOD1Tg/mNOD1KO) and mNOD1KO mice were IP infected with 2.10^7^ M58 *lipl21*- mutant. Bacterial loads were measured by qPCR in blood at 8, 24 and 48 hours post infection. This graph is a compilation of 2 independent experiments, representative of 3 equivalent experiments, with n = 3 to 5 mice in each experiment. (B) hNOD1Tg/mNOD1KO mice were IP infected with 2.10^7^ M58 *lipl21*- mutant or 2.10^7^ complemented C5M58 *lipl21*-/+ mutant. Bacterial loads were measured by qPCR in blood at 8, 24 and 48 hours post infection. The unpaired *t* test was used to compare at each time point the 2 strains. In grey, we indicated the p values that were close to, but did not reach 0.05, the *p* value considered as significant. This experiment is representative of 3 equivalent experiments with n = 3 to 5 in each group, suggesting the same trend.

Since we did not find an altered phenotype related to the LipL21 role in mice or gerbils, and showed that human NOD1 receptors are able to sense leptospiral PG, we next examined whether the LipL21 lipoprotein could be important in the defense against *Leptospira* spp. in humans. As a surrogate of the hNOD1 response, we infected mutant humanized mice expressing a transgene of the human NOD1, in a background of murine NOD1KO mice (hNOD1Tg/mNOD1KO) [21], and mNOD1KO with the M58 *lipl21*- mutant. Although at 8h post infection both NOD1 humanized mice and NOD1KO mice showed the same load of bacteria, the humanized mice harbored significantly fewer leptospires than mNOD1KO at 24h post infection (Fig 6A). Moreover, the infection of NOD1 humanized mice with M58 and the C5M58 complemented strain resulted in a lower bacterial load observed for M58 compared to C5M58 at 48h and 72h post infection (Fig 6B). Although these results did not reach statistical significance, this trend suggests a slight better clearance of the M58 strain compared to the C5M58 expressing LipL21, which is in contrast with the results showing no difference of clearance of M58 and C5M58 in WT mice (Fig 5A). These data suggest that the sensing of M58, devoid of LipL21, by human NOD1 is responsible for the increased clearance.

Together these results indicate that LipL21 is important for protection of PG from detection by the NOD receptors, and therefore may help *Leptospira* spp. to escape the immune responses of some hosts, such as humans.

## Discussion

In this work, we show that pathogenic leptospires avoid the innate immune response mediated by cytosolic NOD receptors through close association of the LipL21 lipoprotein with PG, thereby blocking the release of muropeptides. Moreover, a second layer of protection against murine NOD1 recognition is conferred by the peculiar composition of the leptospiral peptidoglycan, which is almost devoid of GM4, the preferential agonist of murine NOD1 [20].

Cytosolic NOD1 and NOD2 receptors recognize invasive bacteria, such as *Shigella flexneri*, present within the cytosol of host cells. In addition, NOD receptors are able to sense invasive intracellular bacteria residing in vacuoles, such as *Salmonella enterica* and also extracellular pathogens such as *Helicobacter pylori* that can both secrete muropeptides into the host’s cytosol using type III or type IV secretion systems, respectively [26, 27]. *L*. *interrogans* are usually thought of as extracellular bacteria and genomic studies have not identified Type III nor Type IV secretion systems [28]. However, several studies have suggested intracellular survival of pathogenic leptospires in mouse or human macrophages [29, 30], as well as in zebra fish macrophage-like cells [31], indicating that muropeptides of pathogenic leptospires could indeed be in contact with the cytosolic NOD sensors. Moreover, free muropeptides can either directly enter cells through specific transporters, such as the human intestinal peptide transporter 1, (hPEPT1) for MDP [32], or through phagocytosis of GM3 that is further processed to di- or tri-peptides and actively transported by the hPEPT2 in the cytosol [33]. Therefore, all bacteria either pathogenic or commensal can be detected by the NOD receptors.

Different types of PG modifications are known to impair NOD1 recognition, such as a change of the third amino acid in the stem peptide (*meso*DAP). This is the case for *Borrelia* spp. that have an ornithine instead of a *meso*DAP residue. The amidation of the second or third residue of the peptide stem is another mechanism that impairs the NOD1 recognition in *Staphylococcus aureus* and *Lactobacillus plantarum*. Modifications of the glycan chains are known to confer lysozyme resistance, such as O-acetylation of the MurNAc in *S*. *aureus* and in *Listeria monocytogenes* [34], or N-glycolylation of the MurNAc in *Mycobacterium tuberculosis* (reviewed in [35]). We have previously shown that N-deacetylation of the GlcNAc moiety conferred lysozyme resistance in *Listeria monocytogenes* [34, 36]. Therefore, to our knowledge, the 2 mechanisms found in *L*. *interrogans* of Lip21 binding on the PG and the near-absence of GM4 muropeptides have not been previously described and constitute novel bacterial strategies to escape the immune response. Moreover, for the first time we assigned a function to the leptospiral lipoprotein, LipL21. Indeed leptospires, like other spirochetes, are characterized by having many lipoproteins awaiting functional characterization [37].

The lack of difference in bacterial loads between WT and NOD1/2DKO mice upon infection with a virulent strain of *L*. *interrogans* strain L495 drove us to study the potentially atypical composition of leptospiral PG. Despite improved digestion in absence of LipL21 and the use of optimized protocols adapted to a large number of PGs from Gram-positive and Gram-negative bacteria, we never succeeded in completely digesting leptospiral PG [38]. Nevertheless, the HPLC profiles of *L*. *interrogans* muropeptides are roughly similar to those described for the PG composition of the saprophytic *L*. *biflexa* Patoc strain [15]. Indeed, HPLC digestion profiles revealed that although *L*. *biflexa*’s PG conforms to a Gram-negative chemotype, a small proportion of modified muropeptides were rarely observed in other bacteria, such as dimers lacking one of the GlcNAc groups or dimers amidated on the *meso*DAP residue [15]. As in *L*. *biflexa*, we found that *L*. *interrogans* harbors modified deacetylated muropeptides, suggesting that leptospires possess peculiar enzymes such as deacetylases. Despite treatment with various enzymes, such as trypsin and chemotrypsin, to remove typical protein contaminants in preparing the PG, the amount of digested PG was always very limited compared to *E*. *coli*, and did not allow for efficient mass spectrometry analysis. This suggests that, in addition to LipL21, other mechanisms or specificities of pathogenic leptospiral PG hinder the digestion with mutanolysin, an enzyme with the same specificity as lysozyme, cleaving between the two sugars. Hence, the study of the hydrolases of *Leptospira* spp. could be of interest to better understand the unique structure and functions of leptospiral PG.

According to the *meso*DAP composition of the leptospiral PG [16], it was expected that the leptospiral muropeptides could be recognized by hNOD1 and to a lesser extent by hNOD2 [16]. The amount of MDP present in the PG was too small to be detected by HPLC, but we found that it was released by *L*. *interrogans* PG, using HEK293T reporter cells expressing the hNOD2 receptor. The HPLC profile found for the *L*. *interrogans* PG, with only GM3, no GM5 and only minimal amounts of GM4, seems unusual since it suggests that *L*. *interrogans* rapidly processes its muropeptides to trim them in GM3. This is frequently the case for Gram-positive bacteria such as *Staphylococcus carnosus* [39], whereas Gram-negative bacteria usually tend to keep the muropeptides as GM4. Therefore, near-absence of GM4 is a modification of PG that to our knowledge has never been shown for other bacteria with cell walls associated with an outer membraner. However, HPLC profiles of muropeptides are available for a restricted number of bacterial spp. and this phenomenon may be more frequent than previously thought. These results suggest that the reduction of the amount of GM4 muropeptide could be an adaptation of pathogenic *Leptospira* spp. to avoid the murine NOD1 recognition. This would be very important because mice are, like rats, prominent reservoirs of leptospires. It would be interesting to identify the L-D peptidase responsible for the cleavage of the stem peptide. Interestingly, published data about the PG of the saprophytic strain *L*. *biflexa* strain Patoc showed a small peak of GM4 [15], suggesting that the PG of *L*. *biflexa* strain Patoc could be recognized by the murine NOD1. Indeed, using the reporter cell system, we showed that purified PG of the Patoc strain was detected *in vitro* by the murine NOD1. However, this strain was equally cleared by the WT and NOD1/2DKO mice, suggesting that *in vivo*, the NOD receptors did not recognize this saprophytic strain. Likewise for the GM2, the NOD2 agonist, the HPLC profile of muropeptides of the Patoc strain did not reveal a peak of GM4, most probably because of the poor efficiency of PG digestion. We looked for, but did not identify, proteins associated with the PG of the Patoc strain that could have impaired the *in vivo* recognition by mNOD1. We speculate that the distant homolog of LipL21 found in Patoc, may play the same role of PG binding, with a lower affinity for PG, explaining why we did not find it attached to the PG after SDS boiling. Further biochemical studies are required to study the potential role and affinity of the Patoc’s LipL21 to the PG. It is difficult to understand why the PG from this saprophytic strain would also avoid the NOD immune system. However, because the Patoc strain is sensitive to complement, which is a highly efficient mechanism to destroy bacteria, we cannot exclude that the NOD response may have been masked.

The species-specificity of murine NOD1 versus human NOD1 towards leptospires is reminiscent to the species specificity of TLR4, with human TLR4 unable to recognize the LPS from *L*. *interrogans* while murine TLR4 recognizes it [5]. We have previously shown that the TLR4 response is crucial to defense against *L*. *interrogans* in mice [7]. Nevertheless, *L*. *interrogans* can overcome this response and colonize the kidney [10, 40]. We have also shown that in mice NOD1 is a potent PRR in the kidney, actively participating in the control of experimental infection with uropathogenic *E*. *coli* through neutrophil recruitment [41]. We can speculate that NOD1 recognition, if active in mice, would not have allowed the kidney colonization by *L*. *interrogans*. By contrast, leptospires would not need to escape the recognition by NOD1 in humans because they avoid TLR4 recognition of their atypical LPS [5], potentially resulting in severe infection. The systematic study of species specificity of TLR and NOD receptors in different mammalian hosts towards the leptospiral MAMPs would most probably provide insights concerning the host susceptibility and characteristics of leptospirosis.

The fact that 30 min boiling in SDS was not enough to obtain a leptospiral PG sacculus prone to digestion is compelling. It suggests that the interaction between the PG and its “contaminant” is very strong, although not covalent, as it detaches after 4 hour of boiling in SDS. Moreover, the fact that repeated freeze/thaw cycles led to an increased digestion of the PG suggested that the interacting partner could be a protein, compatible with our finding of the LipL21 lipoprotein being associated with the PG. Interestingly, the only leptospiral peptides identified by mass spectroscopy from proteins released from the PG were those of LipL21. These results suggest that the leptospiral PG specifically binds to LipL21 and not to other lipoproteins colocalized with the PG layer, such as LipL32 or Loa22. The lack of co-identification of the Loa22 lipoprotein was surprising, since it has roughly the same molecular weight than LipL21, it is anchored in the outer membrane and possesses a large OmpA domain, known as a peptidoglycan-binding domain. However we did not find Loa22 associated to the peptidoglycan after SDS boiling. The finding that heterologous expression of LipL21 in *E*. *coli* reproduced the effect of LipL21 on *E*. *coli* PG indicates that the phenotype that we observed in leptospires is actually due to LipL21 rather than minor contaminants. In addition, our results show that the lipid anchor of the LipL21 lipoprotein is not required for the binding to PG, because we removed the LipL21 signal sequence including the lipobox sequence [37] to replace it by the signal peptide of OmpA, which is not a lipoprotein. Although recombinant LipL21 had previously been expressed and purified from *E*. *coli* [22], we did not obtain soluble cytosolic LipL21 despite number of trials using different expressing vectors and *E*. *coli* strains. One explanation could be linked to the presence of several cysteine residues in LipL21 that cannot form disulfide bridges in the cytoplasm. Expression of LipL21 transported in the periplasm of *E*. *coli* through its own signal peptide was not toxic when it was fused with alkaline phosphatase at the C-terminal part of the protein. This suggests that the non-anchored C-terminal part of the lipoprotein may be required for the binding to PG, or that a change of conformation because of the fusion impaired the binding. Interestingly, although the muropeptide composition is rather different between *E*. *coli* and *L*. *interrogans*, the binding of LipL21 still occurs to both types of PGs suggesting that a common feature might be key to recognition such as the *meso*DAP or the sugar backbone.

Among different bacterial proteins known to bind the PG are 2 lipoproteins: the peptidoglycan associated lipoprotein (PAL), which binds non-covalently to PG, and the Braun’s lipoprotein (Lpp), a very abundant small lipoprotein of *Escherichia coli*, which is covalently linked to the *meso*DAP residue of the PG peptide stem. Both play a role in anchoring the PG to the inner leaflet of the outer membrane (reviewed in [42]). However, we did not find any DNA or protein sequence homology between the LipL21 from *L*. *interrogans* and Lpp or PAL. Moreover, in *Leptospira* spp. the PG has been shown to be associated with the inner membrane [37], suggesting of a different functional role of LipL21 compared to those lipoproteins. However, LipL21 has been shown to be localized in the outer leaflet of the outer membrane [22]. It is difficult to reconcile the present results with these data, as the size of LipL21 would not allow it to be anchored in the outer leaflet of the outer membrane and at the same time linked to PG. One possibility is that LipL21 behaves as the Lpp, which has recently being shown to coexist in two forms, one residing in the periplasm linked to the PG through the C-term Lysine and another free in the outer membrane [43].

Both PG-binding proteins Lpp and PALs, as well as Loa22, are required for virulence [42] [44]. Accordingly, we showed that the *lipL21-* mutant M58 is avirulent in mice and gerbils. Although we showed that the lack of virulence of the M58 mutant in mice is not due to the NOD sensing, nor to an increased susceptibility to lysozyme, we did not check whether the loss of virulence could be due to an enhanced recognition by other PRRs of the peptidoglycan recognition receptors (PGRPs) family. Indeed, PGRPs are expressed in a wide range of mammalian cells, bind the PG and have an important bactericidal activity [45]. The leptospiral field remains hampered by the difficulty to achieve homologous recombination for clean on site complementation [46]. The fact that complementation, which properly restored expression of LipL21, did not restore the virulence of M58 could be due to the new mutation introduced by the random insertion of the transposon carrying the *lipl21* gene. Also, we don’t know whether the lack of regulation of expression of the complemented *lipl21 in trans*, is important for its function. Therefore, obtaining new *lipl21* mutants and complemented strains would be useful in clarifying the role of LipL21 in virulence. If not involved in virulence, LipL21 would join LipL32, the major leptospiral lipoprotein, as extremely well conserved lipoproteins in pathogenic strains that are expressed in hosts, yet whose absence strikingly does not impair virulence in mice, rats, and hamsters [47]. Given the role of LipL21 in the protection of PG and immune escape, and its leptospiral-specific distribution among all three sub-groups (pathogenic, intermediate pathogenic and non-pathogenic), it suggests a similar role of LipL21 in all *Leptospira* spp. It is possible that LipL21 could have different capacities to protect the PG, with a stronger association with PG in pathogenic leptospires and a weaker association in the non-pathogenic subgroup.

Importantly, we showed that LipL21 could be effective in impairing the human NOD1 recognition, using a humanized mouse model expressing the human NOD1. Our results suggest that LipL21, although not required in mice since leptospiral PG is not recognized by the murine NOD1 and very poorly by NOD2, would be important in humans to block the NOD1 sensing.

To conclude, we found no evidence of any role of NOD receptors in sensing leptospires and therefore the origin of inflammation observed in the TLR2/4DKO mice is not due to NOD activation. This total lack of NOD recognition is surprising since to our knowledge all the pathogenic bacteria yet studied, either intracellular or extracellular, are recognized by one or the other or both of the NOD receptors. The consequences of *L*. *interrogans* escape from recognition by NOD receptors will be an important subject of future studies.

## Materials and methods

### Leptospiral strains and culture conditions

*L*. *biflexa* sevorar Patoc strain Patoc (Paris) and bioluminescent derivative PFlum7, *L*. *interrogans* Icterohaemorraghiae strain Verdun, *L*. *interrogans* serovar Copenhageni strain Fiocruz L1-130, L. *interrogans* serovar Manilae strain L495 and bioluminescent derivative strain MFLum1 were described earlier [7, 9, 10]. Bacteria were grown in Ellinghausen-McCullough-Johnson-Harris (EMJH) medium (Bio-Rad) at 28°C without agitation.

### Generation of *lipl21*^-^ and complemented *L*. *interrogans* mutants

Transposon mutagenesis was performed on *L*. *interrogans* serovar Manilae strain L495 as previously described and the location of the transposon insertion was determined by direct sequencing of genomic DNA [48]. One mutant (M58) obtained using the *Himar 1* transposon with a kanamycin resistant cassette was localized in the 3’ part end of the LA_0011 (LMANv2_100108 | +2 | 972824–973384) gene, corresponding to the LipL21 lipoprotein (*lipL21*^-^). A second transposon mutagenesis using a spectinomycin resistance cassette was performed on the *lipl21*^-^ mutant to reintroduce the *lipl21* gene under the control of its native promoter. The complemented strain C5M58 was called *lipl21*^*-/+*^. The localization of the second transposon of *lipl21*^-/+^was in the hypothetical protein LA_0094.

LipL21 expression was checked by immunoblotting experiments using the polyclonal anti-rabbit serum directed against the LipL21 protein diluted 1:10000, as described [22].

### Infection experiments with leptospires

Male and female C57BL/6J mice (7- to 10-week old) were used in this study and were from Janvier (Le Genest, France). FVB mice were from Charles Rivers Laboratory. LysM knock–out (KO) mice in a FVB background, mice deficient for NOD1 (mNOD1KO), transgenic mice expressing the human NOD1 and deficient for the murine NOD1, named hNOD1Tg/mNOD1KO [21], mice deficient for both NOD1 and NOD2 (NOD1/2DKO), all in a C57BL6/J background, were raised at the Institut Pasteur animal facility and were previously described [40]. Female gerbils (4-week old) were from Janvier.

Infections with *L*. *interrogans* serovar Manilae strains were conducted as described [10]. Just before infection, bacteria in late exponential phase (around 5.10^8^ leptospires *per* ml) were centrifuged at room temperature for 25 min at 3250 ×g, resuspended in endotoxin-free PBS, and counted using a Petroff-Hauser chamber. Leptospires in 200 μl of PBS were injected via the intraperitoneal route (IP) into mice. Most experiments were done with a dose of 2.10^7^ or less corresponding to a sublethal dose of the pathogenic *L*. *interrogans* Manilae. Higher doses up to 5.10^8^ bacteria, provoking a lethal or severe infection were only used for determination of the susceptibility of NOD1/2DKO mice to leptospires. Gerbils were infected with 10^6^ *L*. *interrogans*/ml in EMJH through the IP route. In some experiments, the virulent Manilae bioluminescent MFlum1 strain was used as control since it has been obtained by random insertion of a transposon cassette, like the *lipl21*^-^ mutant [10].

### Bioluminescence imaging

Imaging was performed as described [10]. Because the dark fur of C57BL/6 mice partly blocks the emission of light, mice were shaved on the back the day before imaging. Ten minutes before imaging, 100 μl of D-luciferin potassium salt (Caliper Life Sciences, 30 mg/ml in PBS), the substrate of Firefly luciferase, was intraperitoneally injected to mice. Mice were anesthetized using a constant flow of 2.5% isoflurane mixed with oxygen and air as recommended by the manufacturer, using an XGI-8 anesthesia induction chamber (Xenogen Corp.). Mice were maintained in the anesthesia chamber for at least 5 min to allow adequate dissemination of the injected substrate. Bacterial infection images were acquired using an IVIS Spectrum system (Xenogen Corp., Alameda, CA) according to instructions from the manufacturer. Analysis and acquisition were performed using Living Image 3.1 software (Xenogen Corp.). Images were acquired using the automatic mode (acute phase) or 5 min of integration time (chronic phase and pictures) with a binning of 8 and with the emission filter in the “open” mode. All other parameters were held constant. Quantification was performed using a region of interest defined manually (kidneys) and the results were expressed as photons (P) per second (s) per cm^2^ per steradian (SR).

### Leptospiral loads

The leptospiral burden in blood and urine was determined by quantitative real-time PCR (qPCR), as described [40]. The Maxwell 16 automat was used to extract total DNA from 50 μl of blood and from a drop of urine, using the Maxwell blood DNA and cell LEV DNA purification kits (Promega), respectively. Primers and probe designed in the *lpxA* gene of *L*. *interrogans* strain Fiocruz L1-130 [4] were used to specifically detect pathogenic *Leptospira* spp. [40]. qPCR reactions were run on a Step one Plus real-time PCR apparatus using the absolute quantification program (Applied Biosystems), with the following conditions for FAM TAMRA probes: 50°C for 2 min, 95°C for 10 min, followed by 40 cycles with denaturation at 95°C for 15 s and annealing temperature 60°C for 1 min, according to the manufacturer’s instructions.

### Purification of peptidoglycan and HPLC analysis of muropeptides

Leptospiral peptidoglycan (PG) was purified from late-exponential-phase culture as previously described [38]. Two different times, 0.5 h [38] and 4 h [15], of boiling incubation in 4% SDS were used to extract the PG. PG at a final concentration of 6 mg/mL in endotoxic free water, arising from the two different extractions were called respectively PG 0.5 h and PG 4 h. PG (250 to 500 μg) was digested with 100 U of mutanolysin from *Streptomyces globulosporus* (Sigma) in 12.5 mM sodium phosphate buffer (pH 5.6) for 16h at 37°C as described [38]. The reaction was stopped by boiling for 2 min. To reduce sugar moieties, sodium borohydride (10 mg/mL final) was added to the soluble muropeptide fraction in a 0.5 M sodium borate buffer (pH 9.0). After 15 min of incubation, the pH was adjusted to 2 with orthophosphoric acid to stop the reaction. Muropeptides were analyzed by high-performance liquid chromatography (HPLC) as described previously [38] with a Hypersil reversed-phase octyldecyl silane (C18) column (4.6 x 250 mm, flow-rate of 0.5 ml/min; ThermoHypersil-Keystone) at 52°C. Muropeptides are detected at a 206 nm wavelength using a Shimadzu SPD-20A-UV-Vis detector. To confirm muropeptide structures by mass spectrometry, an additional digestion of the PG prepared with the 4 h protocol was performed with chemotrypsin as described [15] to remove all protein contaminants. Individual muropeptides peaks were collected as they eluted from the HPLC column, then desalted and analyzed by LC-MS as described previously [38, 49].

### NF-κB luciferase assay of muropeptide recognition by NOD proteins

Human epithelial cell line HEK293T (ATCC-CRL-3216), which does not express NOD2 and only minimal amounts of human NOD1, was transfected with a NF-κB-luciferase reporter construct together with NOD1 or NOD2 and ß-galactosidase expressing vectors as described [38]. Control muropeptides (100 nM final) from Invivogen (MurTriDAP as NOD1 agonist, MDP as NOD2 agonist, and FK156 as murine NOD1 agonist [50]) or PG, that cannot enter the cells, have been co-transfected. The activities of luciferase and ß-galactosidase were measured from cellular lysates in the presence of their substrates, using a luminometer and ELISA reader, respectively. Data are expressed as mean ± SD of triplicate values of light units, normalized to the ß-galactosidase activity.

### Identification of LipL21 by mass spectrometry

LipL21 identity assignment on coomassie-stained 4–15% gels was confirmed by mass spectrometry (MS). MS analyzes were carried out by the Plateforme de Biophysique Moléculaire at the Institut Pasteur. Coomassie stained 21 kDa gel bands were excised using a robotic workstation ProPic Investigator. Each sample was digested by trypsin and analyzed by MALDI TOF-MS5 as previously described [51].

### LipL21 cloning in *E*. *coli*

Cloning of full-length *lipl21* in pRSF-duet and in fusion with alkaline phosphatase, as well as LipL21 devoid of its own signal sequence in pASK-IBA6, have been described in supplementary information material and methods.

### Alkaline phosphatase assays

All plasmids (pILL2156-*FL-phoA*, pILL2156-Δ(2–22)*phoA*, pILL2156-*lipl21-phoA* and pILL2156- ΔN-*lipL21-phoA*) were individually transformed into an MG1655Δ*phoA* strain (*phoA748(del)*::*kan* strain, CGSC, The *Coli Genetic Stock Center*, Yale). Strains were grown in the presence of chloramphenicol (25 μg/mL). Alkaline phosphatase assays were performed as described [52] either on plates or in liquid medium. Briefly, in the first case, 5 μL of an overnight preculture of each transformed strain was spread over a section of an LB plate containing IPTG and 5-Bromo-4-chloro-3-indolyl phosphate (XP), 0.1 mM and 40 μg/mL respectively. Plates were incubated overnight at 37°C and visualized. In the second case, 5 mL of LB medium was seeded with 50 μL of an overnight preculture, and then grown to an OD_600 nm_ of 1.0 in the presence of IPTG (0.5 mM). XP was added to a final concentration of 40 μg/mL and bacteria were incubated for 30 min to 1 h at 37°C with shaking (170 rpm) until a blue coloration appeared. Strains containing pILL2156-*FL-phoA* were used as a positive control, while strains transformed with pILL2156-Δ(2–22)*phoA* were used as a negative control.

Immunodetection of LipL21 was performed according to a standard protocol using a rabbit anti-serum elicited against LipL21 [22] and a secondary antibody (anti-rabbit whole IgG coupled to horseradish peroxidase, elicited in Donkey, Amersham), both diluted 1:10000, and revealed with TMB.

### Lysozyme susceptibility testing

Broth microdilution testing was performed using 96-well plates as previously described [53]. Lysozyme at a final concentration of 2 mg/mL, with and without EDTA (2 mM final concentration) was prepared in 100 μl per well of EMJH medium in a sterile 96-well plate. 100 μl of leptospiral suspension diluted in EMJH medium was added to each well to obtain a final concentration of 2.10^6^ cells/mL. The plate was mixed and incubated at 28°C without agitation for 10 min to 2 hours. After PBS washes, bacteria were incubated in EMJH medium at 30°C for 5 days without agitation. Then 20 μl of 10-fold-concentrated AlamarBlue, a cell growth indicator dye, was added to the wells. AlamarBlue turns from dark blue to bright pink in response to chemical reduction of the growth medium in the presence of viable bacteria. The plate was further incubated at 30°C for 2 days in dark without agitation, and the bacterial growth was observed by the color change of the indicator.

### Genomic sequencing and analysis

Next-generation sequencing was performed on genomic DNA of the L495 parental and M58 strains by the Mutualized Platform for Microbiology (P2M) at Institut Pasteur, using the Nextera XT DNA Library Preparation kit (Illumina), the NextSeq 500 sequencing systems (Illumina), and the CLC Genomics Workbench 9 software (Qiagen) for analysis. Sequence reads were aligned with the sequenced and annotated *L*. *interrogans* serovar Manilae L495 genome(http://www.genoscope.cns.fr/agc/microscope/search/export.php?format=genbank&option=none&S_id=5570) by using the Burrows-Wheeler Alignment tool (BWA mem 0.7.5a) [54]. SNP calling was done with the Genome Analysis Toolkit (GATK 2.7–2) Unified Genotyper [55] by following Broad Institute best practices. To validate the call, candidate SNPs were further filtered by requiring coverage of greater than half of the genome mean coverage and 95% read agreement. All data were submitted to EMBL-EBI. A project was created in order to group M58 reads (ERR2192021) and L495 assembly (ERZ480214) associated to the genomes sequenced in this study and is available by using this accession number: PRJEB23342. (http://www.ebi.ac.uk/ena/data/view/PRJEB23342)

### Ethics statement

All protocols were reviewed by the Institut Pasteur (Paris, France), the competent authority, for compliance with the French and European regulations on Animal Welfare and with Public Health Service recommendations. This project has been reviewed and approved (# 2013–0034 and #2015–0026 to CW) and (#2016–0019 to MP) by the Institut Pasteur ethic committee for animal experimentation (Comité d’éthique d’expérimentation animale CETEA #89), agreed by the French Ministery of Agriculture.

### Statistical analysis

Statistical analysis was performed using Graph Pad Prism software. The unpaired *t* test, (two-tailed P values) was used to compare two groups at the same time point. Values are expressed as mean ± standard error of the mean (SEM). A *p* value < 0.05 was considered significant. *p* values: *p < 0.05, **p < 0.01, ***p < 0.001.

## Supporting information

S1 Text Supplementary material and methods. Cloning of full length LipL21 in *E*. *coli*. Cloning of LipL21 devoid of its own signal sequence in *E*. *coli*. LipL21 cloning for alkaline phosphatase assays. Table S1. Primer list for cloning, PCR checking and sequencing (5’- 3’). Table S2. Vectors list.
(DOCX)

S1 Fig HPLC separation profiles of leptospiral muropeptides. (A)(B) Peptidoglycans, extracted after 0.5 h or 4 h of SDS boiling, obtained from *L*. *interrogans* Verdun, Manilae L495 and Fiocruz L1-130 strains were analyzed by HPLC, after mutanolysin digestion. (A) Freeze/thaw (F/T) cycles of the PG favors its digestion. F/T PG from Fiocruz 0.5 h corresponds to Fiocruz PG, extracted with the 0.5 h protocol, which has been frozen and defrost several times. (B) Equivalent HPLC profiles of PGs 4h of the Verdun and Manilae strains. Commercial *E*. *coli* PG (Sigma) was used as the positive control for mutanolysin digestion and as a reference for the position of the GM4 peak. Mutanolysin alone was used as the negative control. (C) HPLC spectrum of *L*. *interrogans* Manilae L495 and Fiocruz L1-130 muropeptides. Both bacteria show similar profiles, with a major peak of GM3, a small peak of GM3 deacetylated (GM-Ac), and dimers of GM3-GM4. For this experiment, the PGs were treated with chemotrypsin before digestion with mutanolysin, and major peaks collected and analyzed by mass spectrometry. (D) HPLC spectrum of *L*. biflexa PG compared to *L*. *interrogans* PG. *E*. *coli* PG is used as a reference for the position of the GM3 and GM4 peaks.
(TIF)

S2 Fig Clearance of the *L*. *biflexa* Patoc strain is equivalent in WT and NOD1/2DKO mice. Tracking by live imaging after intraperitonal injection with 5.10^8^ *L*. *biflexa* Patoc bioluminescent derivative PFLum7. The imaging has been performed at 30 min, 2 h, 5h, 24h and 48 h post inoculation on anesthetized mice after luciferin administration. The upper graph represents the mean ± SEM of the average radiance of n = 5 mice in each group, imaged in ventral position, and gated on the whole body.
(TIF)

S3 Fig LipL21 lipoprotein phylogenetic tree. Neighbor-joining phylogenetic tree based on leptospiral lipoprotein *lipL21* sequence analysis from 22 pathogenic leptospires, 4 intermediate pathogenic leptospires, 4 non-pathogenic leptospires and 1 *Leptonema illini*. The tree was rooted with *lipL21* sequence of *Leptonema illini*. Accession numbers are shown in parentheses.
(TIF)

S4 Fig Control of LipL21 expression. Immunodetection of whole bacterial extracts using an anti-LipL21 serum to show that the fusion protein of full length LipL21 with the alkaline phosphatase is properly expressed.
(TIF)
