# Supplementary material for: LipL21 lipoprotein binding to peptidoglycan enables Leptospira interrogans to escape NOD1 and NOD2 recognition
Source: PLoS Pathog. 2017 Dec 6;13(12):e1006725. doi: 10.1371/journal.ppat.1006725 (PMC5764436; doi:10.1371/journal.ppat.1006725)
Supplement: S1 Text — Cloning of full length LipL21 in E. coli. Cloning of LipL21 devoid of its own signal sequence in E. coli. LipL21 cloning for alkaline phosphatase assays. Table S1. Primer list for cloning, PCR checking and sequencing (5’- 3’). Table S2. Vectors list. (DOCX) [file ppat.1006725.s001.docx]

**Supplementary Material and Methods**

**Cloning of full length LipL21 in *E. coli***

The *lipl21* gene was amplified from *L. interrogans* Fiocruz DNA using Lipl21NdeIF and Lipl21XhoIR primers (Table S1), ligated into pRSFDuet-1 (Novagen, Kan^R^) using NdeI and XhoI restriction sites (MCS2) (Table S2). The construct/plasmid was transformed in *E. coli* BL21(DE3)-Rosetta-2 (BL21 Rosetta-2) competent cells (Novagen), grown in LB medium supplemented of 0,5 % glucose.

**Cloning of LipL21 devoid of its own signal sequence in *E. coli***

The *lipl21* gene was amplified from *L. interrogans* Fiocruz DNA using L21F and L21R primers (Table 1) and cloned into the multiple cloning site of the vector pASK-IBA6 (IBA LifeScience, Amp^R^). The construct was transformed in *E. coli* DH5-α. Clones were selected using LB Ampicillin (100 µg/mL), checked by PCR amplification and sequenced (Table 1). Protein expression was carried out in the *E. coli* strain BL21 Rosetta-2 carrying the pRARE plasmid (Chloramphenicol ^R^). The clones were selected on LB Ampicillin (100 µg/mL) and Chloramphenicol (25 µg/mL) plates. To express LipL21 in *E. coli*, overnight cultures were used to inoculate LB-Amp-Cm medium to an optical density at 600 nm (OD_600 nm_) of 0.05 and grown under agitation (150 rpm) at 37 °C. Induction was performed following the supplier’s instructions. Briefly, when cultures reached an OD_600nm_ of 0.5, anhydrous-Tetracyclin (a-Tc) (200 µg/L) was added to the cultures and further incubated for 3 hours at 37 °C (180 rpm). Then, bacteria were chilled on ice, harvested by centrifugation (3200 g, 20 min, 4 °C) and washed once with sterile PBS. Subsequently, bacterial pellets were stored at –80 °C until used for peptidoglycan purification. Additionally, a pellet from 1 mL of each culture (before and after induction) was kept at – 20°C to further check the expression of LipL21.

**Lipl21 cloning for alkaline phosphatase assays**

The *lipL21* gene was amplified from *L. interrogans* strain Fiocruz L1-130 genomic DNA using oligonucleotides (lip#1-lip#2 and lip3#-lip#2, Table 1), yielding the full-length *lipl21* gene (FL-*lipl21*) and the ΔN-*lipL21* fragment, in which the first 48 base pairs (16 aminoacids), corresponding to the N-terminal putative lipoprotein targeting sequence were removed. In both cases, the stop codon was removed. The alkaline phosphatase (AP) gene (*phoA*) was amplified from *E.* coli MG1655 genomic DNA with oligonucleotides pho#1 and pho#2 or pho#1B and pho#2 to yield two PCR products: one corresponding to the full-length AP gene (FL-*phoA*) and the other to a fragment in which the first 63 base pairs (2-22 first amino acid positions) corresponding to the periplasmic targeting sequence were removed (Δ(2-22)*phoA*). In a second step, both *lipl21* fragments (FL-*lipl21* and ΔN-*lipL21*) and Δ(2-22)*phoA* were digested with the appropriate restriction enzymes (see Table S1) and fused together by ligation and introduced into the pILL2156 shuttle vector (chloramphenicol resistance gene) (Table S2) between *Nde*I and *Bam*HI restriction sites to obtain FL-*lipl21-phoA* and ΔN-*lipL21-phoA* in-frame fusions. *FL-phoA* and Δ(2-22)*phoA* fragments were also individually cloned into pILL2156 plasmid between *Nde*I and *Bam*HI restriction sites for control vectors.

**Table S1. Primer list for cloning, PCR checking and sequencing (5’- 3’)**

| *Primer* | *Sequence (5' to 3')* | *Site* | *Sens* | *Comments* |
| --- | --- | --- | --- | --- |
| Lipl21NdeIF | AAAGGG**CATATG**ATCAATAGACTTATAG | NdeI | Fwd | FL-LipL21 cloning in pRSF-Duet1 |
| Lipl21XhoIR | TTTGGG**CTCGAG**TTATTGTTTGGAAACCTC | XhoI | Rev | FL-LipL21 cloning in pRSF-Duet1 |
| lip#1 | AAAGGG**CATATG**ATCAATAGACTTATAG | NdeI | Fwd | FL-LipL21 |
| lip#2 | TTT**GGTACC**TTGTTTGGAAACCTCTT | KpnI | Rev | FL and ΔN-LipL21  (no stop codon) |
| lip#3 | AAAGGG**CATATG**GCTTGTTCCAGTACT | NdeI | Fwd | ΔN-LipL21 |
| pho#1 | ACCCGGG**CATATG**AAACAAAGCACTATTGCAC | NdeI | Fwd | FL phoA (prophoA) |
| pho#2 | TTT**GGATCC**TTATTTCAGCCCCAGAGCGGC | BamHI | Rev | FL and  ∆(2-22)phoA |
| pho#1B | TTTA**GGTACC**ATGACACCAGAAATGCCTGTTCTG | KpnI | Fwd | ∆(2-22)phoA  (3'-fusion with *lipL21*) |
| pho#1C | ACCCGGG**CATATG**ACACCAGAAATGCCTGTTCTG | NdeI | Fwd | ∆(2-22)phoA  (alone in pILL2157) |
| L21F | A**CCGCGG**ATGTTCCAGTACTGACACAGG | SacII | Fwd | ∆N_1-17_-LipL21 cloning |
| L21R | GCT**CCATGG**TTGTTTGGAAACCTCTTGAGC | NcoI | Rev | in pASK-IBA6 |
| LpcrF | GTGGACCTCCAGAGCAAAGA | - | Fwd | PCR check *lipL21* |
| LpcrR | GATCCGGAACCGGTTGCTTT | - | Rev | PCR check *lipL21* |
| seqF | GAGTTATTTTACCACTCCCT | - | Fwd | Sequencing primer pIBA |
| seqR | GAGTTATTTTACCACTCCCT | - | Rev | Sequencing primer pIBA |

Boldface indicates restriction sites; FL: Full-length

**Table S2. Vectors list**

| *pILL2157 vectors* | *Restriction sites* | *Use* |
| --- | --- | --- |
| FL-*lipl21-phoA* | NdeI/KpnI | LipL21 localization |
| ΔN-*lipL21-phoA* | NdeI/KpnI | LipL21 localization |
| ∆(2-22)- *phoA* | NdeI/BamHI | Negative control |
| *prophoA* | NdeI/BamHI | Positive control |
| *pRSFDuet1 vector* | ***Restriction sites*** | ***Use*** |
| *FL-lipl21* | NdeI/XhoI | LipL21 expression |
| *pASK-IBA vector* | ***Restriction sites*** | ***Use*** |
| *pASK-IBA-6* | SacII/NcoI | Lip21 expression |
