# Supplementary figures and images for: LipL21 lipoprotein binding to peptidoglycan enables Leptospira interrogans to escape NOD1 and NOD2 recognition
Source: PLoS Pathog. 2017 Dec 6;13(12):e1006725. doi: 10.1371/journal.ppat.1006725 (PMC5764436; doi:10.1371/journal.ppat.1006725)

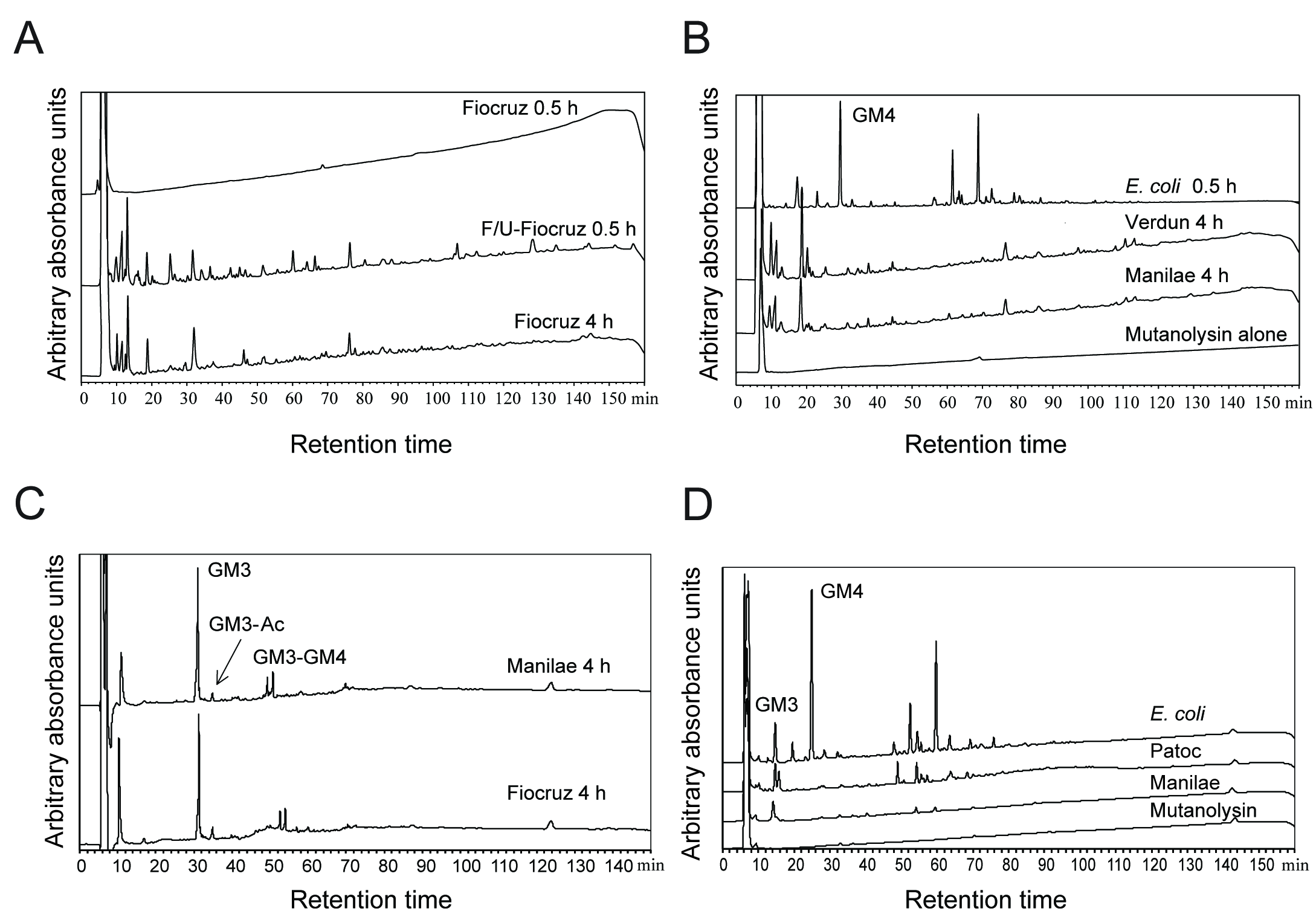

Supplement: S1 Fig — (A)(B) Peptidoglycans, extracted after 0.5 h or 4 h of SDS boiling, obtained from L. interrogans Verdun, Manilae L495 and Fiocruz L1-130 strains were analyzed by HPLC, after mutanolysin digestion. (A) Freeze/thaw (F/T) cycles of the PG favors its digestion. F/T PG from Fiocruz 0.5 h corresponds to Fiocruz PG, extracted with the 0.5 h protocol, which has been frozen and defrost several times. (B) Equivalent HPLC profiles of PGs 4h of the Verdun and Manilae strains. Commercial E. coli PG (Sigma) was used as the positive control for mutanolysin digestion and as a reference for the position of the GM4 peak. Mutanolysin alone was used as the negative control. (C) HPLC spectrum of L. interrogans Manilae L495 and Fiocruz L1-130 muropeptides. Both bacteria show similar profiles, with a major peak of GM3, a small peak of GM3 deacetylated (GM-Ac), and dimers of GM3-GM4. For this experiment, the PGs were treated with chemotrypsin before digestion with mutanolysin, and major peaks collected and analyzed by mass spectrometry. (D) HPLC spectrum of L. biflexa PG compared to L. interrogans PG. E. coli PG is used as a reference for the position of the GM3 and GM4 peaks. (TIF) [file ppat.1006725.s002.tif]

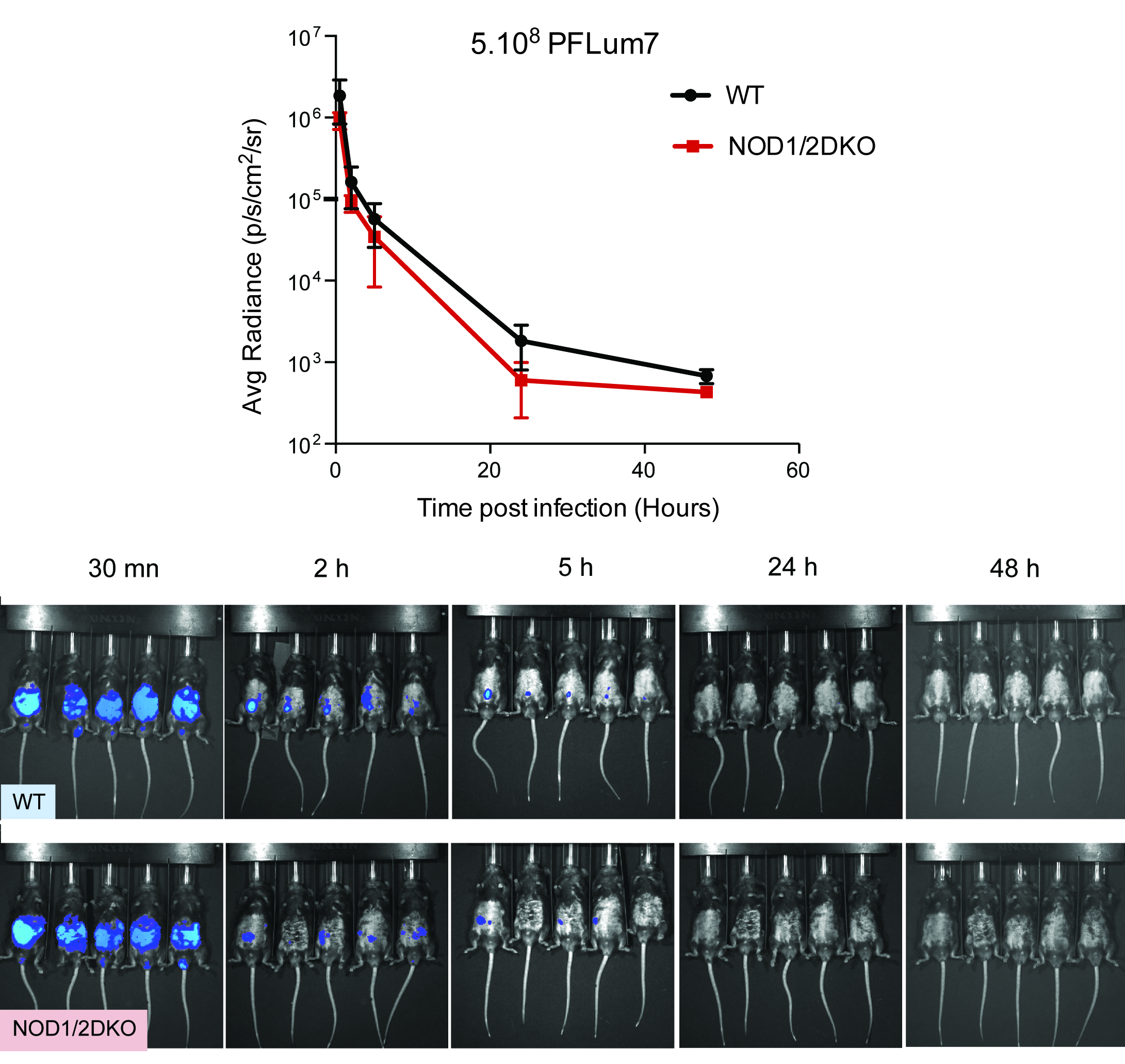

Supplement: S2 Fig — Tracking by live imaging after intraperitonal injection with 5.108 L. biflexa Patoc bioluminescent derivative PFLum7. The imaging has been performed at 30 min, 2 h, 5h, 24h and 48 h post inoculation on anesthetized mice after luciferin administration. The upper graph represents the mean ± SEM of the average radiance of n = 5 mice in each group, imaged in ventral position, and gated on the whole body. (TIF) [file ppat.1006725.s003.tif]

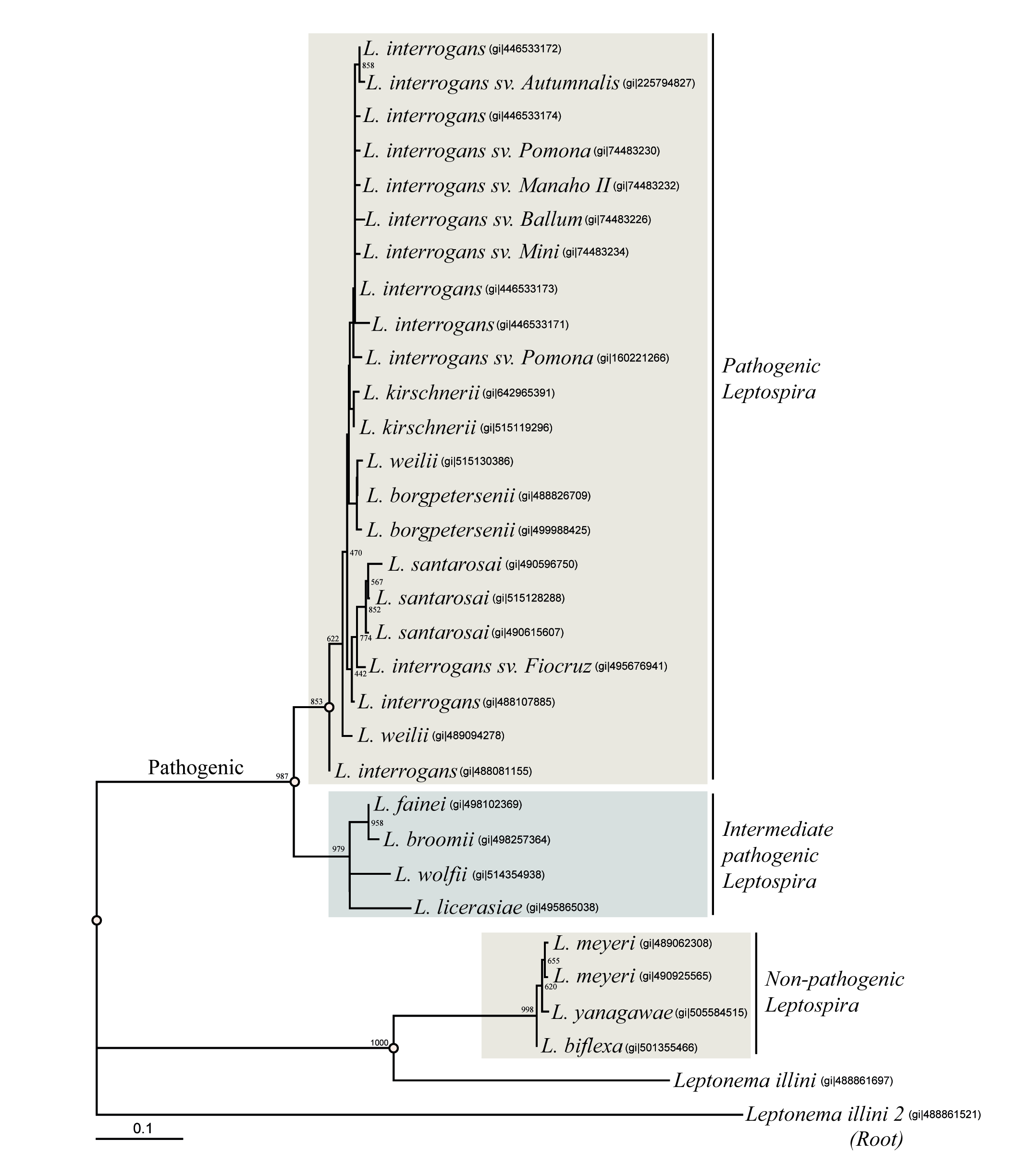

Supplement: S3 Fig — Neighbor-joining phylogenetic tree based on leptospiral lipoprotein lipL21 sequence analysis from 22 pathogenic leptospires, 4 intermediate pathogenic leptospires, 4 non-pathogenic leptospires and 1 Leptonema illini. The tree was rooted with lipL21 sequence of Leptonema illini. Accession numbers are shown in parentheses. (TIF) [file ppat.1006725.s004.tif]

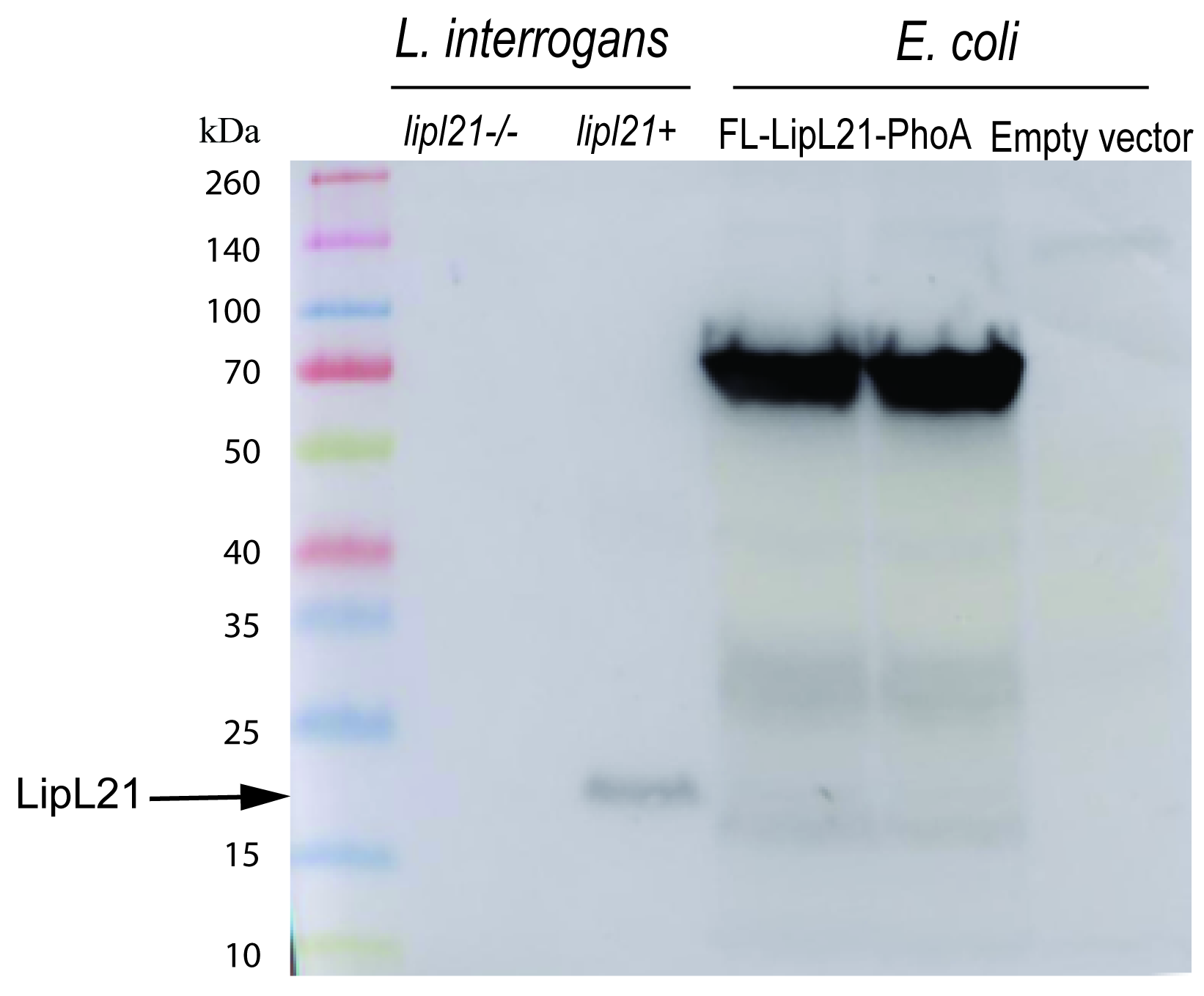

Supplement: S4 Fig — Immunodetection of whole bacterial extracts using an anti-LipL21 serum to show that the fusion protein of full length LipL21 with the alkaline phosphatase is properly expressed. (TIF) [file ppat.1006725.s005.tif]
